# The burden and epidemiology of orofacial clefts in Iran from 1990 to 2023: a Global Burden of Disease study with subnational socio-demographic index analysis

**DOI:** 10.1371/journal.pone.0352804

**Published:** 2026-07-08

**Authors:** Reza Sharifi, Seyed Aria Nejadghaderi, Farnoosh Mohammadi, Mohammad Javad Kharazifard, Zeinab Bakhtiari

**Affiliations:** 1 Craniomaxillofacial Research Center, Tehran University of Medical Sciences, Tehran, Iran; 2 Department of Oral and Maxillofacial Surgery, Dental School, Tehran University of Medical Sciences, Tehran, Iran; 3 HIV/STI Surveillance Research Center, and WHO Collaborating Center for HIV Surveillance, Institute for Futures Studies in Health, Kerman University of Medical Sciences, Kerman, Iran; 4 Knowledge Hub for Migrant and Refugee Health, Institute for Futures Studies in Health, Kerman University of Medical Sciences, Kerman, Iran; 5 Dental Research Center, Dentistry Research Institute, Tehran University of Medical Sciences, Tehran, Iran; 6 Oral Maxillofacial Surgery Resident, Department of Oral and Maxillofacial Surgery, Dental School, Tehran University of Medical Sciences, Tehran, Iran; Karolinska Institutet, SWEDEN

## Abstract

**Background:**

Orofacial clefts (OFCs) are among the most frequent congenital craniofacial malformations worldwide. These conditions impose lifelong functional, psychological, and socioeconomic burdens. In Iran, despite several hospital-based and regional studies, an updated national and subnational assessment of OFC burden has been lacking. This study aimed to estimate the incidence, prevalence, disability-adjusted life years (DALYs), and mortality attributable to OFCs in Iran and its 31 provinces from 1990 to 2023, and to explore their associations with the sociodemographic index (SDI).

**Methods:**

Data were obtained from the Global Burden of Disease (GBD) Study 2023. OFCs were defined according to ICD-10 codes Q35–Q37. GBD modeled estimates were generated using the DisMod-MR 2.1 meta-regression framework. The DisMod-MR 2.1 meta-regression model was applied to derive estimates of metrics. Rates were age-standardized per 100,000 population, and 95% uncertainty intervals (UIs) were calculated. The relationship between OFC burden and SDI was assessed descriptively using smoothing splines models.

**Results:**

In 2023, Iran recorded 1,208.9 (917.1–1,581.4) new OFC cases, corresponding to an age-standardized incidence rate of 2.5 per 100,000. Prevalence reached 77.3 (62.3–94.7) per 100,000, and DALYs were 6.9 (4.1–10.5) per 100,000. Since 1990, DALY and mortality rates decreased by 88.8% and 96.1%, respectively, while prevalence remained largely stable. The highest prevalence was seen in Tehran (108.3 per 100,000) and the lowest in Isfahan (49.1 per 100,000). The greatest DALY burden occurred among children under five years. There was a negative relationship between SDI and DALY rates.

**Conclusions:**

The national burden of OFCs in Iran has substantially declined over the past three decades. These findings highlight the need for sustained efforts to improve cleft prevention, early detection, and access to specialized care to further reduce the burden and promote health equity across Iran.

## Introduction

Craniofacial anomalies are among the most common congenital malformations worldwide and are associated with lifelong functional, psychosocial, and health-system burdens on affected individuals and families [1]. Orofacial clefts (OFCs), described as cleft lip with or without cleft palate and isolated cleft palate, are the most frequent and visible craniofacial defects, requiring coordinated surgical, dental, speech, and psychosocial care from infancy through adulthood [2]. These subtypes differ in embryologic origin, clinical severity, timing of intervention, and long-term functional outcomes, which makes them important to consider in epidemiologic studies.

The etiology of non-syndromic OFCs is complex and multifactorial, arising from interactions among different genetic loci and a range of maternal and environmental exposures [2]. Several maternal factors have been associated with increased cleft risk, including active and passive maternal smoking, abnormal maternal body mass index, pre-existing diabetes and hypertension, and certain medication or environmental exposures during the periconceptional period [3].

Global burden estimates indicated that in 2021 there were about 4.1 million people living with OFCs worldwide, with an age-standardized prevalence of approximately 53.4 per 100,000 and an age-standardized incidence near 3.0 per 100,000; although mortality attributable to clefts was low in most settings, the age-standardized DALY rate was estimated at about 5.8 per 100,000, with regional variation that concentrated burden in low- and middle-income regions [4]. In Iran, studies have shown non-negligible prevalence and important geographic variations. National prevalence was estimated as 1.0–1.3 per 1000 live births with higher rates reported in some provinces and urban centers such as Tehran [5]. In addition, local studies have reported differences in subtype distribution, sex ratios, and access to rehabilitative services, underscoring the need for reliable subnational surveillance and trend analysis [6,7].

Although prior studies reported the burden of OFCs at the global and regional levels using the burden of disease data [4,8–10], important knowledge gaps remain for Iran. Most previous GBD-based analyses have been global or regional, and few have provided a long-term, province-level assessment for a specific country with marked geographic heterogeneity. In particular, no recent study has comprehensively described the OFC burden in Iran across both national and subnational levels over a 34-year period. Providing recent and updated data for health policymakers is important for decision making. In this study, we used the most recent global burden of disease (GBD) 2023 study to report the burden of OFCs in Iran and its provinces from 1990 to 2023.

## Methods

### Overview

This study used data from the GBD Study 2023, a comprehensive project led by the Institute for Health Metrics and Evaluation to quantify global health loss. The GBD 2023 provides a global health audit by estimating the burden of 375 diseases and injuries and 88 associated risk factors, covering 204 countries and territories, including 660 subnational locations, from 1990 to 2023 [11,12]. For this study, we focused on the burden of OFCs within Iran and its provinces. We extracted the finalized GBD 2023 estimates and conducted a descriptive secondary analysis of these pre-generated outputs. No new primary DisMod-MR 2.1 modeling was performed in this study. We extracted the finalized GBD 2023 estimates for incidence, prevalence, mortality, years of life lost (YLLs), years lived with disability (YLDs), and DALYs for Iran and its 31 provinces, and then summarized these outputs for the present analysis. The ethics committee of Tehran University of Medical Sciences approved the present study (ethics code: IR.TUMS.DENTISTRY.REC.1404.096).

### Defining the condition

OFCs are defined to include isolated cleft lip, isolated cleft palate, and the combined cleft lip and cleft palate, all resulting from the developmental failure of facial tissues to properly fuse. The GBD case definition specifically captures isolated cleft palate (ICD-10 codes: Q35.2–Q35.9 except for Q35.4) and cleft palate with or without cleft lip (ICD-10 codes: Q36.0, Q36.1, Q36.9, Q37.1, Q37.5, Q37.8, and Q37.9), while excluding craniofacial clefts that do not involve the oropharynx. Although surgical treatment is the typical course of action, mostly occurring early in life, the long-term impact is measured by three sequelae: disfigurement level 1, disfigurement level 2, and disfigurement level 2 with speech problems. To account for all affected individuals, a proportion of the population is classified as asymptomatic, and in the absence of more granular data, an equal distribution among these sequelae was assumed [11]. Although OFC phenotypes differ in biological and clinical characteristics, they were analyzed as a combined category because this is how the GBD 2023 outputs were available for Iran and its provinces. This grouping improves comparability across years and locations, but it reduces clinical granularity and limits phenotype-specific interpretation and real-world applicability of the burden estimates. The equal-distribution assumption among sequelae was used within the GBD framework when more granular sequela-specific data were unavailable, and we acknowledge that this simplifying assumption may influence DALY estimation.

### Mathematical modeling

We did not independently re-fit the full GBD model. Instead, we extracted and analyzed the finalized GBD 2023 estimates as a descriptive secondary analysis. The disease burden of OFCs was estimated using the Disease Modelling Meta-Regression version 2.1 (DisMod-MR 2.1) model. The prevalence component included random effects limited to ±0.8 to restrain geographical variation in the estimated birth prevalence. The model accounted for surgical repair, or remission, by setting it to zero for the first three months of life. Remission was then capped at a maximum of 0.8 for ages three months to two years, aligning with the period when up to 75% of cases are repaired, before being reduced for older age groups. It was set to zero for ages 50 and older. Similarly, the excess mortality rate was constrained by maximum prior values: 2.5 for the early neonatal period, 0.01 for ages 5–10 years, and 0.000001 for ages 10 years and older, reflecting the highest risk of mortality in the first few years of life [11]. These GBD assumptions should be interpreted as model-based constraints rather than direct measurements from Iran, and therefore the estimates are best viewed as ecological population summaries.

### Measuring health loss

Health loss was quantified using three interconnected metrics. Cause-specific mortality estimates were based on the GBD 2023 cause of death model. YLLs were calculated by multiplying the number of deaths by a standard life expectancy at each age. YLDs were determined by multiplying the condition’s prevalence by its associated disability weight. The overall disease burden, disability-adjusted life years (DALYs), was derived as the sum of YLLs and YLDs. All reported estimates, including incidence, prevalence, death, and DALYs, were presented as counts and age-standardized rates per 100,000 population. Also, the 95% Uncertainty Intervals (UIs) were calculated from 500 estimation draws, using the 2.5th and 97.5th percentiles of the resulting distribution [11]. All estimates were extracted in a standardized format to ensure consistency across provinces, years, sex, and age groups.

### Analysis and contextualizing of findings

The relationship with the SDI was assessed using Smoothing Splines models. The SDI is a composite developmental index, scaled from 0 (least developed) to 1 (most developed). The SDI has three components, including per capita income, average years of schooling for those aged 15 and older, and the fertility rate in women aged 25 and younger. The statistical analysis was carried out using R software (version 4.5.1). For this analysis, SDI was treated as a continuous predictor and age-standardized OFC burden metrics were used as outcomes to explore potential non-linear patterns across provinces and over time. Spline curves were fitted in R using standard smoothing spline procedures, with the smoothing parameter selected automatically by generalized cross-validation, allowing the algorithm to balance goodness of fit against model complexity and reduce the risk of overfitting. No degrees of freedom or smoothing parameters were manually specified or optimized, because the analysis was intended to provide descriptive visualization rather than formal statistical inference or prediction. Accordingly, no hypothesis testing or model-based causal interpretation was performed, and the observed SDI–burden relationships should be interpreted as population-level ecological patterns rather than individual-level associations.

## Results

### Burden of OFC in Iran, the world, and the Middle East and North Africa region

In 2023, the estimated incident cases of OFCs in Iran were 1,208.9 (95% UI: 917.1 to 1,581.4), corresponding to an age-standardized incidence rate of 2.5 per 100,000 population (95% UI: 1.9 to 3.3). From 1990 to 2023, the incidence rate decreased by 38.8% (95% UI: −48.2 to −28.5). Also, the total prevalent cases in Iran were estimated at 66,851.0 (95% UI: 53,624.7 to 82,163.0), with an age-standardized rate of 77.3 per 100,000 population (95% UI: 62.3 to 94.7). The national rate of prevalence had a small and non-significant decrease of 4.9% (95% UI: −13.0 to 3.9) between 1990 and 2023. The total DALYs attributed to OFCs in 2023 were 5,136.6 (95% UI: 3,233.4 to 7,565.0), with an age-standardized rate of 6.9 per 100,000 population (95% UI: 4.1 to 10.5). There was also a decrease in the DALY rate by 88.8% (95% UI: −95.8 to −62.8) from 1990 to 2023. OFCs resulted in 12.1 deaths (95% UI: 3.5 to 27.4) in 2023. The death rates had a decline of 96.1% (95% UI: −98.9 to −81.7) since 1990 (Table 1 and S1-S4 Figs).

**Table 1:** Prevalent cases, incident cases, deaths, and DALYs, along with their age-standardized rates of orofacial clefts in 2023 for both sexes, and the percentage change in rates per 100,000 population from 1990 to 2023 in Iran.

Location	Incidence (95% UI)			Prevalence (95% UI)			DALYs (95% UI)			Deaths (95% UI)		
	Counts (2023)	Rate (2023)	Pcs in rate 1990-2023	Counts (2023)	Rate (2023)	Pcs in rate 1990-2023	Counts (2023)	Rate (2023)	Pcs in rate 1990-2023	Counts (2023)	Rate (2023)	Pcs in rate 1990-2023
**Global**	180983.1 (130107.4, 236926.2)	3.0 (2.1, 3.9)	−0.2 (−0.3, −0.2)	4256822.4 (3458443.4, 5200871.4)	54.2 (44.1, 66.3)	0.0 (0.0, 0.1)	555613.1 (290997.7, 1136874.6)	8.1 (4.0, 17.5)	−0.8 (−0.9, −0.5)	3273.4 (710.6, 10055.8)	0.1 (0.0, 0.2)	−0.8 (−0.9, −0.5)
**Middle East and North Africa**	16922.5 (12874.1, 22144.7)	3.0 (2.3, 3.9)	−0.4 (−0.5, −0.3)	548883.1 (448921.1, 668754.5)	86.1 (70.4, 104.9)	0.0 (−0.1, 0.0)	73808.1 (36404.1, 190128.4)	12.3 (5.9, 32.7)	−0.7 (−0.8, −0.2)	449.5 (102.4, 1777.0)	0.1 (0.0, 0.3)	−0.8 (−0.9, −0.3)
**Iran (Islamic Republic of)**	1208.9 (917.1, 1581.4)	2.5 (1.9, 3.3)	−38.8 (−48.2, −28.5)	66851.0 (53624.7, 82163.0)	77.3 (62.3, 94.7)	−4.9 (−13.0, 3.9)	5136.6 (3233.4, 7565.0)	6.9 (4.1, 10.5)	−88.8 (−95.8, −62.8)	12.1 (3.5, 27.4)	0.0 (0.0, 0.1)	−96.1 (−98.9, −81.7)
Alborz	30.5 (23.0, 42.4)	2.3 (1.7, 3.2)	−40.9 (−52.0, −28.7)	2181.1 (1730.6, 2732.3)	71.5 (57.2, 89.3)	−15.4 (−24.7, −5.3)	142.2 (87.7, 202.5)	5.1 (3.1, 7.3)	−77.4 (−89.8, −47.3)	0.1 (0.0, 0.3)	0.0 (0.0, 0.0)	−95.5 (−98.9, −82.5)
Ardebil	17.3 (12.9, 23.2)	2.4 (1.8, 3.2)	−46.2 (−57.3, −34.1)	935.6 (752.7, 1160.9)	72.1 (57.8, 89.0)	−15.8 (−25.5, −3.3)	68.1 (42.1, 99.1)	5.9 (3.5, 8.8)	−92.5 (−97.7, −67.4)	0.1 (0.0, 0.3)	0.0 (0.0, 0.0)	−97.9 (−99.5, −85.4)
Bushehr	18.9 (14.0, 25.6)	2.3 (1.7, 3.2)	−47.9 (−57.2, −35.4)	950.9 (746.1, 1178.6)	72.2 (57.0, 89.2)	−15.6 (−24.8, −2.8)	94.5 (53.1, 152.0)	8.9 (4.7, 15.5)	−91.5 (−97.2, −66.7)	0.4 (0.1, 1.0)	0.0 (0.0, 0.1)	−95.5 (−98.9, −79.7)
Chahar Mahaal and Bakhtiari	16.5 (12.3, 22.6)	2.5 (1.9, 3.4)	−48.9 (−58.9, −36.5)	758.2 (598.5, 943.5)	72.6 (57.6, 90.3)	−16.7 (−25.1, −6.7)	64.5 (40.0, 98.2)	7.1 (4.1, 11.6)	−92.7 (−97.6, −71.7)	0.2 (0.0, 0.5)	0.0 (0.0, 0.1)	−97.1 (−99.3, −87.3)
East Azarbayejan	55.2 (42.1, 76.7)	2.7 (2.1, 3.8)	−31.6 (−43.1, −14.2)	3491.3 (2757.0, 4279.1)	84.5 (67.8, 103.3)	17.1 (2.2, 35.5)	248.4 (152.2, 361.0)	6.9 (4.2, 10.1)	−91.1 (−96.7, −67.6)	0.4 (0.1, 0.9)	0.0 (0.0, 0.0)	−97.6 (−99.3, −88.4)
Fars	58.9 (44.6, 79.6)	2.4 (1.8, 3.2)	−41.9 (−51.3, −30.7)	3687.9 (2882.9, 4514.2)	71.8 (56.0, 88.4)	−15.0 (−24.5, −5.3)	274.4 (174.1, 398.6)	6.3 (3.9, 9.5)	−85.3 (−94.9, −52.7)	0.6 (0.2, 1.2)	0.0 (0.0, 0.0)	−94.8 (−98.7, −74.5)
Gilan	22.0 (16.5, 30.1)	2.3 (1.8, 3.2)	−45.8 (−56.0, −34.6)	1842.1 (1455.4, 2307.9)	71.7 (57.1, 89.5)	−15.7 (−25.6, −5.1)	121.5 (76.6, 180.0)	5.5 (3.3, 8.0)	−84.7 (−94.2, −58.0)	0.1 (0.0, 0.3)	0.0 (0.0, 0.0)	−96.3 (−99.1, −84.8)
Golestan	32.0 (23.9, 43.5)	2.5 (1.9, 3.4)	−34.2 (−46.4, −17.1)	1572.2 (1232.5, 1965.8)	78.3 (61.7, 97.8)	18.2 (3.3, 36.1)	112.0 (72.3, 159.0)	6.0 (3.9, 8.6)	−85.5 (−94.5, −52.7)	0.2 (0.1, 0.4)	0.0 (0.0, 0.0)	−96.7 (−99.1, −83.9)
Hamadan	21.1 (16.0, 29.1)	2.4 (1.8, 3.3)	−42.4 (−52.9, −30.2)	1232.0 (975.9, 1531.0)	72.0 (57.9, 88.8)	−14.6 (−23.9, −4.7)	94.9 (60.4, 146.3)	6.6 (4.1, 10.5)	−90.5 (−96.8, −63.9)	0.2 (0.1, 0.5)	0.0 (0.0, 0.1)	−96.5 (−99.1, −77.6)
Hormozgan	36.0 (26.5, 50.9)	2.5 (1.8, 3.5)	−44.4 (−54.4, −30.7)	1489.3 (1180.7, 1847.2)	72.3 (57.5, 89.5)	−15.4 (−23.5, −5.2)	172.5 (91.4, 297.0)	9.9 (4.9, 18.3)	−91.0 (−97.0, −65.5)	0.9 (0.2, 2.4)	0.1 (0.0, 0.2)	−94.8 (−98.6, −75.8)
Ilam	8.2 (6.2, 11.1)	2.3 (1.8, 3.2)	−47.0 (−56.7, −36.3)	445.6 (356.9, 545.6)	71.7 (57.6, 87.5)	−16.4 (−27.0, −6.8)	32.1 (20.2, 47.2)	5.7 (3.6, 8.4)	−88.6 (−96.1, −61.3)	0.1 (0.0, 0.1)	0.0 (0.0, 0.0)	−97.0 (−99.2, −83.0)
Isfahan	43.8 (30.5, 61.1)	1.8 (1.3, 2.6)	−30.4 (−45.3, −12.9)	2634.4 (1932.0, 3441.1)	49.1 (36.1, 63.6)	9.7 (−6.9, 29.6)	246.7 (141.8, 385.9)	6.6 (3.3, 11.8)	−92.6 (−97.3, −69.9)	1.0 (0.2, 2.3)	0.0 (0.0, 0.1)	−95.8 (−98.8, −84.2)
Kerman	47.1 (35.3, 62.7)	2.3 (1.7, 3.1)	−43.9 (−54.9, −30.1)	2513.9 (2030.9, 3109.5)	72.1 (58.2, 89.4)	−14.8 (−24.3, −4.3)	201.3 (124.0, 302.9)	6.7 (4.0, 10.4)	−86.2 (−95.0, −55.8)	0.5 (0.2, 1.2)	0.0 (0.0, 0.1)	−94.7 (−98.5, −73.8)
Kermanshah	24.6 (18.9, 34.1)	2.4 (1.9, 3.4)	−46.1 (−56.7, −32.5)	1428.0 (1135.1, 1757.0)	72.2 (57.7, 88.5)	−15.9 (−25.3, −5.1)	111.7 (70.2, 163.9)	6.8 (4.2, 10.6)	−91.0 (−96.8, −68.6)	0.3 (0.1, 0.6)	0.0 (0.0, 0.1)	−96.6 (−99.0, −82.9)
Khorasan-e-Razavi	107.2 (81.0, 143.8)	2.4 (1.8, 3.2)	−45.0 (−55.8, −33.5)	5060.5 (4071.9, 6207.1)	72.2 (58.2, 88.4)	−15.2 (−25.3, −4.9)	406.3 (241.5, 586.6)	6.5 (3.8, 9.9)	−91.9 (−97.2, −70.4)	1.1 (0.3, 2.3)	0.0 (0.0, 0.1)	−97.2 (−99.3, −85.7)
Khuzestan	85.7 (63.7, 115.0)	2.5 (1.9, 3.4)	−39.7 (−50.4, −26.6)	3677.3 (2948.5, 4555.8)	72.3 (58.4, 89.3)	−14.4 (−22.8, −4.4)	315.9 (187.5, 490.2)	7.0 (4.1, 11.3)	−86.7 (−95.5, −52.8)	1.0 (0.3, 2.5)	0.0 (0.0, 0.1)	−94.4 (−98.8, −72.5)
Kohgiluyeh and Boyer-Ahmad	13.2 (9.9, 17.6)	2.3 (1.8, 3.1)	−47.8 (−56.9, −36.5)	573.1 (457.6, 702.9)	71.7 (57.7, 87.8)	−16.1 (−25.1, −5.3)	62.4 (32.8, 109.8)	9.2 (4.7, 17.4)	−93.8 (−98.1, −69.9)	0.3 (0.0, 0.8)	0.1 (0.0, 0.1)	−96.7 (−99.3, −83.7)
Kurdistan	23.8 (17.8, 31.3)	2.4 (1.8, 3.1)	−46.0 (−55.7, −31.5)	1258.0 (993.6, 1579.6)	72.0 (57.1, 90.1)	−16.7 (−24.7, −6.3)	93.7 (57.8, 134.0)	6.1 (3.6, 8.8)	−92.4 (−97.4, −70.4)	0.2 (0.1, 0.4)	0.0 (0.0, 0.0)	−97.8 (−99.4, −86.2)
Lorestan	24.9 (18.8, 33.4)	2.3 (1.8, 3.1)	−44.4 (−53.9, −32.3)	1280.2 (1015.9, 1555.4)	71.6 (57.4, 86.8)	−15.6 (−23.3, −6.1)	86.8 (55.2, 126.7)	5.2 (3.3, 7.6)	−84.6 (−94.0, −56.1)	0.1 (0.0, 0.2)	0.0 (0.0, 0.0)	−97.1 (−99.2, −85.7)
Markazi	15.3 (11.6, 21.2)	2.4 (1.8, 3.3)	−43.3 (−54.1, −30.2)	1037.2 (820.1, 1277.5)	71.9 (57.1, 88.5)	−15.2 (−23.1, −5.2)	74.5 (47.2, 110.4)	6.1 (3.7, 9.2)	−88.2 (−95.9, −61.9)	0.1 (0.0, 0.3)	0.0 (0.0, 0.0)	−96.3 (−99.0, −83.1)
Mazandaran	33.5 (25.2, 46.2)	2.3 (1.7, 3.2)	−45.4 (−54.7, −35.3)	2517.1 (2022.6, 3079.0)	71.3 (57.4, 86.7)	−16.3 (−24.6, −4.3)	168.6 (107.7, 255.9)	5.5 (3.5, 8.3)	−85.3 (−94.5, −56.8)	0.2 (0.0, 0.4)	0.0 (0.0, 0.0)	−96.5 (−99.1, −84.8)
North Khorasan	14.1 (10.7, 19.2)	2.5 (1.9, 3.3)	−42.1 (−53.1, −28.8)	629.6 (495.7, 781.5)	72.4 (57.1, 89.6)	−14.0 (−24.5, −2.5)	45.5 (28.8, 66.2)	5.6 (3.4, 8.4)	−88.5 (−96.2, −59.2)	0.1 (0.0, 0.2)	0.0 (0.0, 0.0)	−97.2 (−99.3, −84.5)
Qazvin	16.5 (12.4, 21.8)	2.4 (1.8, 3.2)	−39.6 (−50.6, −26.9)	978.4 (775.5, 1220.8)	72.3 (57.5, 89.9)	−14.4 (−23.6, −4.4)	70.1 (43.9, 101.9)	5.9 (3.7, 8.8)	−88.1 (−95.8, −60.0)	0.1 (0.0, 0.2)	0.0 (0.0, 0.0)	−96.7 (−99.1, −83.4)
Qom	21.3 (15.7, 29.5)	2.4 (1.8, 3.4)	−40.9 (−52.1, −29.3)	1059.9 (838.3, 1317.1)	72.2 (57.4, 89.6)	−15.2 (−23.9, −4.7)	76.5 (47.9, 113.9)	5.7 (3.5, 8.4)	−87.1 (−95.0, −60.3)	0.1 (0.0, 0.3)	0.0 (0.0, 0.0)	−96.6 (−99.1, −85.8)
Semnan	8.8 (6.8, 12.2)	2.3 (1.8, 3.2)	−44.8 (−54.5, −33.1)	569.4 (453.0, 707.2)	71.7 (57.4, 88.6)	−16.0 (−23.8, −8.3)	43.5 (27.9, 64.5)	6.7 (4.1, 10.3)	−90.3 (−96.3, −67.7)	0.1 (0.0, 0.2)	0.0 (0.0, 0.1)	−96.3 (−99.0, −85.3)
Sistan and Baluchistan	94.5 (71.4, 131.3)	2.5 (1.9, 3.5)	−45.7 (−55.3, −34.6)	2450.4 (1969.6, 2984.4)	72.8 (58.5, 88.8)	−16.3 (−25.4, −6.5)	301.0 (168.0, 518.8)	8.4 (4.7, 14.3)	−89.3 (−95.7, −61.3)	1.7 (0.5, 3.8)	0.0 (0.0, 0.1)	−94.5 (−98.5, −76.1)
South Khorasan	15.9 (12.1, 20.8)	2.3 (1.8, 3.1)	−43.6 (−55.1, −33.2)	667.2 (536.4, 823.6)	71.8 (57.8, 88.5)	−14.6 (−23.4, −5.3)	49.8 (30.5, 72.9)	5.7 (3.5, 8.3)	−88.6 (−96.3, −61.8)	0.1 (0.0, 0.2)	0.0 (0.0, 0.0)	−97.0 (−99.3, −84.1)
Tehran	218.2 (164.7, 297.1)	3.4 (2.6, 4.6)	−13.6 (−28.2, 4.5)	15708.7 (12691.0, 19552.8)	108.3 (88.0, 135.0)	26.2 (11.9, 42.6)	1009.9 (643.2, 1457.4)	7.5 (4.9, 10.9)	−67.3 (−85.3, −21.3)	0.7 (0.2, 1.6)	0.0 (0.0, 0.0)	−94.8 (−98.6, −77.4)
West Azarbayejan	51.6 (38.9, 70.4)	2.4 (1.8, 3.3)	−47.0 (−56.2, −35.1)	2559.3 (2039.3, 3172.5)	71.9 (57.3, 88.7)	−16.4 (−25.1, −6.2)	205.7 (123.2, 311.1)	6.6 (3.9, 10.3)	−92.6 (−97.2, −75.0)	0.6 (0.2, 1.2)	0.0 (0.0, 0.1)	−97.3 (−99.3, −88.4)
Yazd	17.9 (13.5, 24.4)	2.4 (1.8, 3.2)	−44.5 (−53.6, −32.2)	878.3 (689.8, 1089.3)	71.8 (56.5, 88.9)	−15.0 (−23.5, −5.1)	65.1 (39.8, 97.4)	5.9 (3.5, 8.7)	−88.3 (−95.9, −62.7)	0.1 (0.0, 0.3)	0.0 (0.0, 0.0)	−96.6 (−99.1, −83.5)
Zanjan	14.5 (10.9, 19.7)	2.4 (1.8, 3.3)	−50.2 (−60.1, −38.5)	784.0 (618.6, 962.6)	72.0 (57.2, 88.3)	−17.7 (−27.3, −5.5)	76.6 (45.4, 124.6)	9.2 (4.8, 16.3)	−94.8 (−98.4, −79.1)	0.3 (0.1, 0.8)	0.1 (0.0, 0.1)	−97.2 (−99.3, −86.8)

Abbreviations: DALY: disability-adjusted life year; Pcs: percent changes; UI: uncertainty interval. Percent change in rate (abbreviated as “Pcs in rate” in the original table) is calculated as: [(2023 age‑standardized rate – 1990 age‑standardized rate)/ 1990 age‑standardized rate] × 100%. A positive value indicates an increase in the age‑standardized rate over 34 years; a negative value indicates a decrease.

In 2023, the estimated incident cases of OFC worldwide were 180,983.1 (95% UI: 130,107.4 to 236,926.2), corresponding to an age-standardized incidence rate of 3.0 per 100,000 population (95% UI: 2.1 to 3.9). Compared with 1990, the global age-standardized incidence rate showed only a minimal reduction (−0.2%; 95% UI: −0.3 to −0.2). Total prevalent cases globally in 2023 were estimated at 4,256,822.4 (95% UI: 3,458,443.4 to 5,200,871.4), with an age-standardized prevalence rate of 54.2 per 100,000 (95% UI: 44.1 to 66.3). The age-standardized prevalence rate remained stable over the study period. The total DALYs attributable to OFCs in 2023 were 555,613.1 (95% UI: 290,997.7 to 1,136,874.6), with an age-standardized DALY rate of 8.1 per 100,000 (95% UI: 4.0 to 17.5), showing a slight decline of 0.8% per 100,000 (95% UI: −0.9 to −0.5) since 1990 (Table 1 and S1-S4 Figs).

In 2023, the Middle East and North Africa (MENA) region had 16,922.5 incident OFC cases (95% UI: 12,874.1 to 22,144.7), with an age-standardized incidence rate of 3.0 per 100,000 (95% UI: 2.3 to 3.9). The age-standardized incidence rate in MENA decreased by 0.4% between 1990 and 2023 (95% UI: −0.5 to −0.3). Prevalent cases in MENA in 2023 numbered 548,883.1 (95% UI: 448,921.1 to 668,754.5), corresponding to an age-standardized prevalence rate of 86.1 per 100,000 (95% UI: 70.4 to 104.9) with no meaningful changes over the study period. The total DALYs in 2023 were 73,808.1 (95% UI: 36,404.1 to 190,128.4), with an age-standardized DALY rate of 12.3 per 100,000 (95% UI: 5.9 to 32.7), reflecting a decrease of 0.7% per 100,000 (95% UI: −0.8 to −0.2) since 1990 (Table 1 and S1-S4 Figs).

### Burden of OFC in Iran’s Provinces

**Fig 1:**
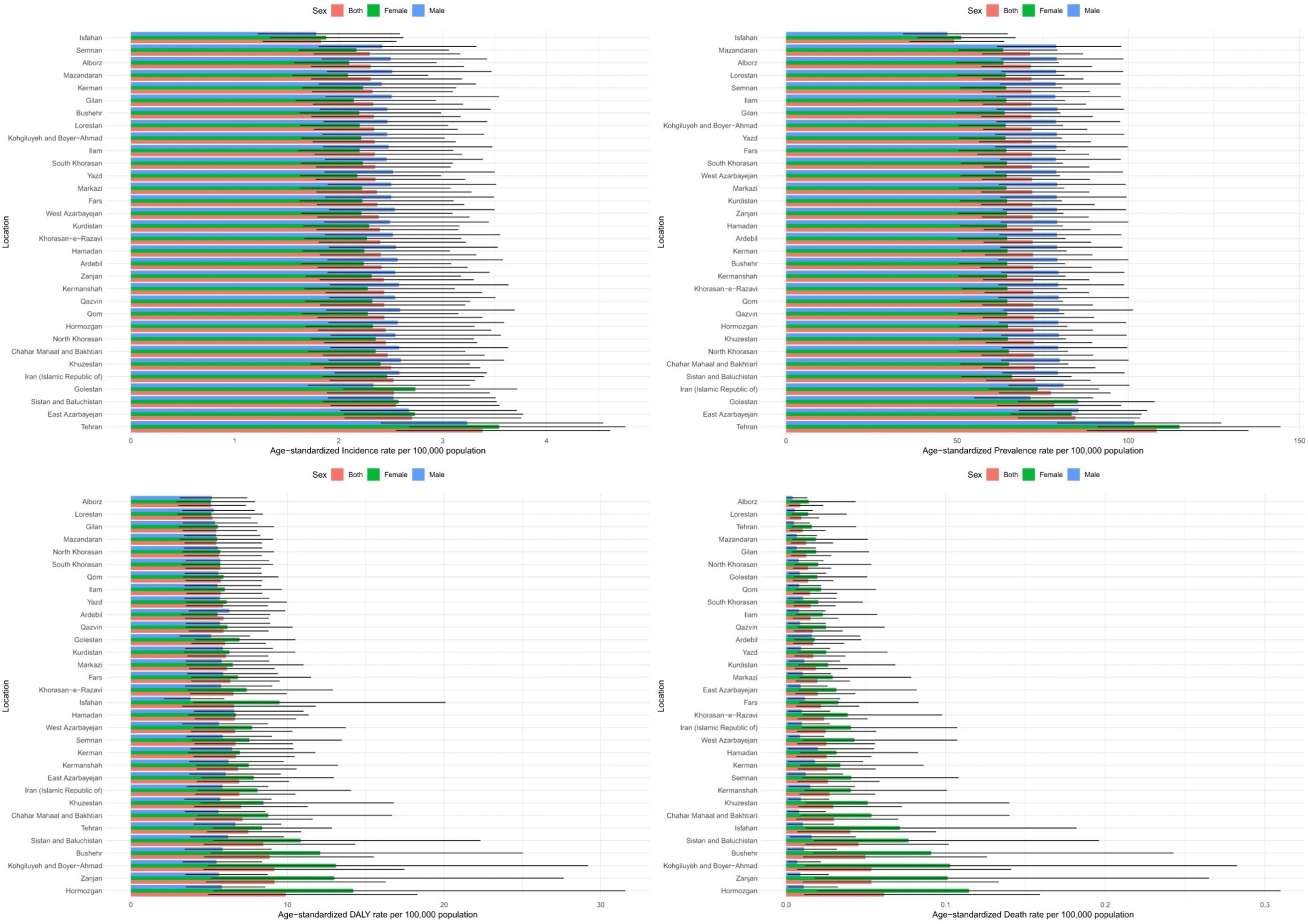
Age-standardized incidence (A), point prevalence (B), DALY (C), and death rates (D) for orofacial cleft (per 100,000 population) in Iran in 2023, by sex and province. DALY = disability-adjusted-life-year. (Generated from data available from http://ghdx.healthdata.org/gbd-results-tool). Bars represent rates per 100,000 population (x-axis) for each location (y-axis), with blue bars indicating males, green bars indicating females, red bars indicating both, and error bars showing 95% uncertainty intervals. Axes units: x-axis (rates per 100,000 population), y-axis (locations ordered by descending rates).

Most provinces had incidence rates hovering around the national average of 2.5 per 100,000. Tehran showed the highest rate at 3.4, while Isfahan had the lowest at 1.8 per 100,000. The largest percentage decrease in incidence rate between 1990 and 2023 was observed in Zanjan (−50.2%), while Tehran had the smallest decrease (−13.6%) (Table 1, Fig 1A, and S5 Fig). These provincial comparisons are descriptive and reflect subnational variation in the GBD estimates and they were not based on formal spatial analysis.

The age-standardized point prevalence in 2023 ranged from a low of 49.1 in Isfahan to a high of 108.3 in Tehran. Only two provinces, Tehran (108.3) and East Azarbayejan (84.5), had rates exceeding the national average of 77.3. Tehran also exhibited the greatest percentage increase in the prevalence rate (26.2%) between 1990 and 2023 (Table 1, Fig 1B, and S6 Fig).

Age-standardized DALY rates had a pattern of significant decrease across all provinces from 1990 to 2023. The lowest rate in 2023 was in Alborz (5.1 per 100,000), and the highest was in Hormozgan (9.9 per 100,000). The percentage change in DALYs rate from 1990 to 2023 showed the largest reductions in Zanjan (−94.8%) and Kohgiluyeh and Boyer-Ahmad (−93.8%), while Tehran had the smallest reduction (−67.3%) (Table 1, Fig 1C, and S7 Fig).

The age-standardized death rate was consistently near zero in all provinces in 2023. Percentage changes in death rates from 1990 to 2023 showed reductions (Table 1, Fig 1D, and S8 Fig).

### Burden of OFC in Iran by age and sex

**Fig 2:**
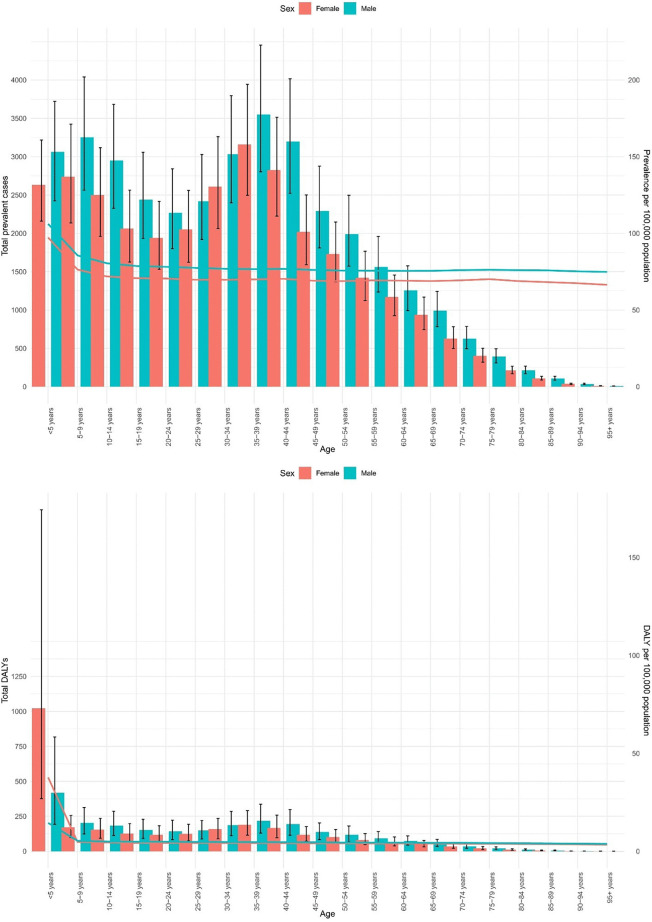
Number of prevalent cases and prevalence (A) and number of DALYs and DALY rate (B) for orofacial cleft (per 100,000 population) in Iran, by age and sex in 2023. DALY = disability-adjusted-life-year. (Generated from data available from http://ghdx.healthdata.org/gbd-results-tool). Lines represent rates for males (blue) and females (red). Bars represent numbers for males (blue) and females (red). Error bars showing 95% uncertainty intervals for numbers.

The highest number of prevalent cases for both sexes occurs in the 35–39 years age group. A high number of prevalent cases was observed through the early and middle adult years, including age groups 30 − 34 years and 40 − 44 years, and is high in young children 5 − 9 years. The number of prevalent cases generally declines sharply after the 55–59 years age group. Also, the < 5 age group had the largest prevalence rate in 2023 in Iran. Males showed a higher total number of prevalent cases than females in almost every age group (Fig 2A).

The < 5 years age group had the greatest DALYs and DALY rate for both males and females. The DALY count sharply declined in the 5 − 9 years age group and remained at a low level throughout the rest of the lifespan. The total number of DALYs was higher for females than males in the < 5 years age group. After the < 5 years age group, males and females had near DALY counts across all other age groups (Fig 2B). This pattern is reported descriptively and should not be interpreted as evidence of a causal sex-specific mechanism.

### Association with socio-demographic index

**Fig 3:**
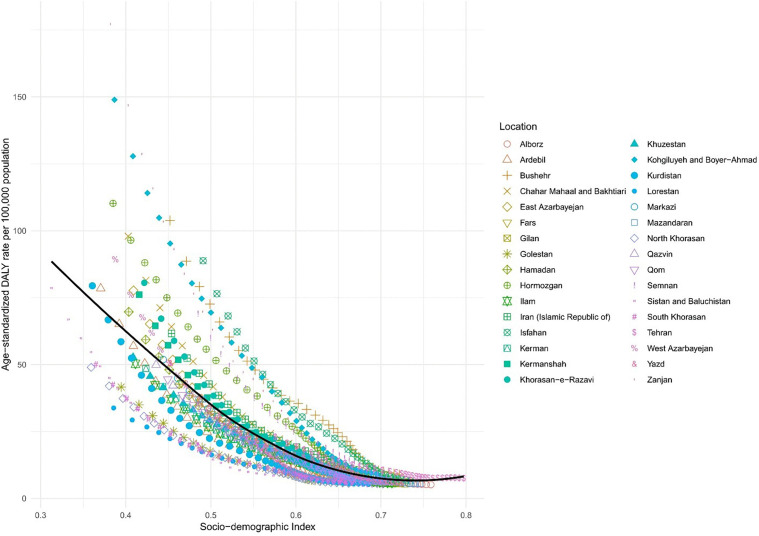
Age-standardized DALY rates of orofacial cleft for the 31 provinces between 1990 and 2023, by SDI; Expected values based on the SDI and the condition rates in all locations are shown as the black line. Each point shows the observed age-standardized DALY rate for each location from 1990 to 2023. DALY = disability-adjusted-life-year. SDI = socio-demographic index. Points are colored by location. Axes units: y-axis (Age-standardized DALY rates per 100,000 population), x-axis (SDI score, 0–1). (Generated from data available from http://ghdx.healthdata.org/gbd-results-tool).

The burden of OFCs, represented by the age-standardized DALY rate per 100,000 population, showed an inverse area-level association with the SDI (Fig 3).

## Discussion

Our study showed a significant decline in age-standardized incidence, DALY, and death rates between 1990 and 2023 in Iran. These trends were accompanied by notable provincial heterogeneity, with Tehran showing the highest age-standardized incidence and prevalence rates while some provinces such as Zanjan experienced the greatest declines. We also found an inverse relationship between OFC burden and the SDI. Because these findings are based on ecological, modeled estimates, they describe population-level patterns rather than individual-level associations.

At the national level, we found that age-standardized incidence rate decreased by 38.8% between 1990 and 2023, and age-standardized DALY rate showed about 89% decrease. These patterns are in accordance with the global patterns. In this regard, the study by Wang et al. showed an estimated annual percent change (EPAC) of −0.69% (95% CI: −0.81 to −0.58) between 1990 and 2019 [13]. Similarly, between 1990 and 2021, there were declining trends for age-standardized DALY rates at both global level (EPAC: −4.13; 95% CI: −4.28, −3.98%) and the MENA region (EPAC: −3.13; 95% CI: −3.41, −2.85%) [4]. Moreover, the results of a systematic review and meta-analysis of 17 articles showed that the prevalence of combined cleft lip and palate was 0.88 per 1,000 births [14]. The larger decline in DALYs observed in Iran compared with global estimates [4] might be partially due to a combination of improving survival, changes in disability patterns, and differences in model-based estimation across geographic levels. However, this interpretation remains cautious because these factors were not directly tested in the present analysis. Also, these should be considered as possible contributing hypotheses rather than confirmed drivers of change [15].

At the provincial level, the observed variation should also be interpreted descriptively. Most provinces had values around the national age-standardized incidence rate, whereas Tehran had both the highest incidence and the highest point prevalence. These differences may be related to variation in case ascertainment, referral concentration, health-service access, and reporting completeness, but these possibilities cannot be confirmed from the current data [16,17]. Tehran’s higher reported rates could partly reflect better case ascertainment in the capital, as well as population movement and urban referral bias, whereas provinces with large declines may have benefited from service access and reporting over time [18]. These explanations are plausible but remain speculative.

By age and sex, our analysis showed a dual pattern. The largest counts of prevalent cases occur in early and middle adulthood, but the greatest DALY burden and highest DALY rates are concentrated in the youngest children, especially those under five years of age. Moreover, males had higher numbers of prevalent cases in most age bands while DALYs in the < 5 group were higher among females. These are in the same way with the findings of the global and regional reports [4,9]. Moreover, the age-standardized DALY rate in the Iran (6.9 per 100,000) was a bit lower than the world (8.1 per 100,000) and the MENA region (12.3 per 100,000). The immediate life-years lost and disability caused by OFCs are concentrated in infancy and early childhood, when feeding, airway, and comorbidity risks are greatest, while prevalent case counts accumulate across the lifespan as treated survivors age. Sex differences in OFC epidemiology have been variably reported, and demographic or subtype composition differences between datasets might explain small sex-specific variations in DALYs or prevalence in Iran relative to other settings [4,8,9]. Accordingly, a study reporting the quality-of-care index found that this index for OFCs improved substantially worldwide between 1990 and 2019 and that gender gaps in quality of care have been narrowing, though low-scoring countries persist [10]. A recent national analysis of live births in Iran over 2016–2021 reported a relatively stable prevalence of cleft lip with/without palate, a persistent male predominance in counts, and clear associations with maternal risk factors, indicating that changes in prevalence may be slow and strongly influenced by modifiable maternal exposures [19]. In the present study, however, these possible determinants were not analyzed, so they should not be interpreted as causal explanations for the observed age- and sex-specific patterns.

We found an inverse association between SDI and age-standardized DALY rates. Similarly, the reports based on the GBD 2021 data showed a similar negative correlation between SDI and age-standardized DALY rates (R = −0.55; p < 0.001) [4]. Moreover, there was a negative correlation between human development index and DALY rate (adjusted beta = −0.05; p = 0.002) in the Eastern Mediterranean Region in 2019 [9]. This ecological association should be interpreted cautiously to avoid ecological fallacy, because province-level SDI does not necessarily represent the socioeconomic conditions of individual patients. As low SDI settings usually face greater barriers to timely diagnosis, neonatal and pediatric care, safe anesthesia and surgical services, and ongoing multidisciplinary rehabilitation, these might explain the higher burden, while further research is necessary to confirm these [20,21]. These barriers are consistent with the pattern observed in our data, but they were not directly measured in this study.

The decline in DALYs and deaths is consistent with a broader improvement in population health and may reflect improved survival, better long-term management, and reduced severity among affected individuals over time, but our study cannot determine the exact reason [22]. In particular, in a congenital condition such as OFC, prevalence may remain stable because treated individuals continue to survive into later age groups, even as mortality and disability burden decline. Moreover, population health gains such as reductions in infant mortality and improved neonatal services can reduce OFC-related morbidity [23]. Similarly, observed provincial inequalities may reflect differences in access, referral, ascertainment, and service availability, but the present analysis does not allow these mechanisms to be distinguished [18,19]. Accordingly, these factors should be considered plausible hypotheses rather than confirmed determinants of the observed trends. Strengthening national centers, training programs for safe pediatric anesthesia and cleft surgery, newborn feeding and airway support protocols, and community outreach for early identification can help reduce provincial inequalities and long-term disability [24].

Our study has some limitations. First, the analysis relied on GBD modeled estimates rather than directly observed registry data, so the results depend on the assumptions and data inputs used in the GBD framework. Although this approach enables standardized comparisons across time and location, it may not fully capture local clinical reality. Second, the GBD case definition and the need to distribute sequelae proportions in the absence of granular local data require simplifying assumptions, which may not perfectly reflect Iran’s clinical reality. Third, we could not evaluate the influence of specific risk factors at the provincial level within the GBD framework, so causal inferences about drivers of observed trends are limited. Fourth, because OFCs were analyzed as a combined category, we could not evaluate subtype-specific patterns for cleft lip with or without palate versus isolated cleft palate. Fifth, the GBD framework does not allow us to distinguish syndromic from non-syndromic OFCs, which limits etiologic interpretation. Sixth, the provincial and SDI analyses are ecological and should not be interpreted at the individual level. Nevertheless, the use of the most recent, internationally comparable GBD 2023 framework to produce a comprehensive, subnational picture of OFC burden for Iran across three decades provides useful context for understanding long-term trends and provincial inequalities. Future studies should compare GBD-derived estimates with Iranian congenital anomaly registry and hospital-based data when such data become available.

## Conclusions

Iran has experienced declines in OFC burden from 1990 to 2023, while national prevalence has remained relatively stable and provincial and age-specific disparities persist. The highest disability burden was among young children and was inversely associated with SDI, highlighting ongoing equity gaps in access to timely and comprehensive cleft care. These findings highlight persistent burden and inequity patterns that may inform future planning for surveillance, registry completeness, diagnosis, treatment access, and follow-up care. More specific prevention and health-system recommendations should be interpreted in light of future studies that directly examine risk factors, care pathways, and service availability.

## Supporting information

S1 Fig Trends of the age-standardized incidence rate of orofacial cleft in the world, Middle East and North Africa, and Iran from 1990 to 2023. (Generated from data available from http://ghdx.healthdata.org/gbd-results-tool).
(TIF)

S2 Fig Trends of the age-standardized prevalence rate of orofacial cleft in the world, Middle East and North Africa, and Iran from 1990 to 2023. (Generated from data available from http://ghdx.healthdata.org/gbd-results-tool).
(TIF)

S3 Fig Trends of the age-standardized disability-adjusted life year rate of orofacial cleft in the world, Middle East and North Africa, and Iran from 1990 to 2023. (Generated from data available from http://ghdx.healthdata.org/gbd-results-tool).
(TIF)

S4 Fig Trends of the age-standardized death rate of orofacial cleft in the world, Middle East and North Africa, and Iran from 1990 to 2023. (Generated from data available from http://ghdx.healthdata.org/gbd-results-tool).
(TIF)

S5 Fig The percentage change in the age-standardized incidence rate of orofacial cleft in Iran from 1990 to 2023, by sex and province. (Generated from data available from http://ghdx.healthdata.org/gbd-results-tool).
(TIF)

S6 Fig The percentage change in the age-standardized point prevalence of orofacial cleft in Iran from 1990 to 2023, by sex and province. (Generated from data available from http://ghdx.healthdata.org/gbd-results-tool).
(TIF)

S7 Fig The percentage change in the age-standardized DALY rates of orofacial cleft in Iran from 1990 to 2023, by sex and province. DALY = disability-adjusted-life-year (Generated from data available from http://ghdx.healthdata.org/gbd-results-tool).
(TIF)

S8 Fig The percentage change in the age-standardized death rates of orofacial cleft in Iran from 1990 to 2023, by sex and province. (Generated from data available from http://ghdx.healthdata.org/gbd-results-tool).
(TIF)

## References

[1] Shkoukani MA, Chen M, Vong A. Cleft lip - a comprehensive review. Front Pediatr. 2013;1:53. doi: 10.3389/fped.2013.00053 24400297 PMC3873527

[2] Rahimov F, Jugessur A, Murray JC. Genetics of nonsyndromic orofacial clefts. Cleft Palate Craniofac J. 2012;49(1):73–91. doi: 10.1597/10-178 21545302 PMC3437188

[3] Ács M, Cavalcante BGN, Bănărescu M, Wenning AS, Hegyi P, Szabó B, et al. Maternal factors increase risk of orofacial cleft: a meta-analysis. Sci Rep. 2024;14(1):28104. doi: 10.1038/s41598-024-79346-7 39548204 PMC11568291

[4] Wang Z, Qi W, Chen Y, Niu F. Global, regional, and national burden of orofacial clefts, 1990–2021: an analysis of data from the global burden of disease study 2021. Frontiers in Medicine. 2025;12. 10.3389/fmed.2025.1609700 PMC12187660 40568198

[5] Khazaei S, Shirani AM, Khazaei M, Najafi F. Incidence of cleft lip and palate in Iran. A meta-analysis. Saudi Med J. 2011;32(4):390–3. doi: 10.15537/1658-3175.5276 21483999

[6] Farshidfar N, Ajami S, Sahmeddini S, Goli A, Foroutan HR. Epidemiological and Spatiotemporal Descriptive Analysis of Patients with Nonsyndromic Cleft Lip and/or Palate: A 12-Year Retrospective Study in Southern Iran. Biomed Res Int. 2023;2023:7624875. doi: 10.1155/2023/7624875 37124932 PMC10132907

[7] Jalilevand N, Jalaie S. Prevalence of cleft lip and palate among four provinces in the West and North-West of Iran. J Res Med Sci. 2015;20(6):548–53. doi: 10.4103/1735-1995.165951 26600829 PMC4621648

[8] Ma Q, Wei J, Peng B, Liu J, Mo S. Burden of orofacial clefts from 1990-2021 at global, regional, and national levels. Front Pediatr. 2025;13:1502877. doi: 10.3389/fped.2025.1502877 40191646 PMC11968431

[9] Nabavizadeh SS, Mootz JJ, Nadjmi N, Massenburg BB, Khoshnood K, Shojaeefard E, et al. Gender inequality and burden of orofacial clefts in the Eastern Mediterranean region: findings from global burden of disease study 1990-2019. BMC Pediatr. 2024;24(1):76. doi: 10.1186/s12887-024-04569-6 38262976 PMC10804627

[10] Sofi-Mahmudi A, Shamsoddin E, Khademioore S, Khazaei Y, Vahdati A, Tovani-Palone MR. Global, regional, and national survey on burden and Quality of Care Index (QCI) of orofacial clefts: Global burden of disease systematic analysis 1990-2019. PLoS One. 2025;20(1):e0317267. doi: 10.1371/journal.pone.0317267 39774532 PMC11709310

[11] Hay SI, Ong KL, Santomauro DF, A B, Aalipour MA, Aalruz H, et al. Burden of 375 diseases and injuries, risk-attributable burden of 88 risk factors, and healthy life expectancy in 204 countries and territories, including 660 subnational locations, 1990–2023: a systematic analysis for the Global Burden of Disease Study 2023. The Lancet. 2025;406(10513):1873–922. 10.1016/S0140-6736(25)01637-X PMC12535840 41092926

[12] Naghavi M, Kyu HH, A B, Aalipour MA, Aalruz H, Ababneh HS, et al. Global burden of 292 causes of death in 204 countries and territories and 660 subnational locations, 1990–2023: a systematic analysis for the Global Burden of Disease Study 2023. The Lancet. 2025;406(10513):1811–72. 10.1016/S0140-6736(25)01917-8 PMC12535838 41092928

[13] Wang D, Zhang B, Zhang Q, Wu Y. Global, regional and national burden of orofacial clefts from 1990 to 2019: an analysis of the Global Burden of Disease Study 2019. Ann Med. 2023;55(1):2215540. doi: 10.1080/07853890.2023.2215540 37232757 PMC10228319

[14] Haseli A, Hajimirzaie S, Bagheri L, Sadeghian A, Ahmadnia E. Prevalence of Cleft Lip and Cleft Palate in Iran: A Systematic Review and Meta-Analysis. Journal of Mazandaran University of Medical Sciences. 2019;28(168):185–97.

[15] Marandi SA, Farrokhzad N, Moradi R, Rezaeizadeh G, Shariat M, Nayeri FS. Review of the Iranian Newborns’ Health, Survival, and Care and Future Challenges. Arch Iran Med. 2019;22(7):403–9. 31679384

[16] Yokobori Y, Obara H, Sugiura Y, Kitamura T. Gaps in the civil registration and vital statistics systems of low- and middle-income countries and the health sector’s role in improving the situation. Glob Health Med. 2021;3(4):243–5. doi: 10.35772/ghm.2020.01103 34532606 PMC8403260

[17] Biroudian S, Emami AS, Keleshteri ZK, Delpasand K. Prenatal Screening Tests from the Perspective of Medical Ethics and Law: A Qualitative Research in Iran. Fertility, Gynecology and Andrology. 2025;5(5):e158613.

[18] Kianifar H, Hasanzadeh N, Jahanbin A, Ezzati A, Kianifar H. Cleft lip and Palate: A 30-year Epidemiologic Study in North-East of Iran. Iran J Otorhinolaryngol. 2015;27(78):35–41. 25745610 PMC4344973

[19] Heydari M-H, Sadeghian A, Khadivi G, Mustafa HJ, Javinani A, Nadjmi N, et al. Prevalence, trend, and associated risk factors for cleft lip with/without cleft palate: a national study on live births from 2016 to 2021. BMC Oral Health. 2024;24(1):36. doi: 10.1186/s12903-023-03797-z 38185687 PMC10771673

[20] Adeyemi K, Chang V, Ifeanacho E, Sunner R. Socioemotional Development of Children with Cleft Lip With or Without Cleft Palate (CL/P) Across Socioeconomic Backgrounds and Potential Impacts on Families: Narrative Review. The CHILD Journal. 2025;4(1):9–15. doi: 10.15173/child.v4i1.3956

[21] Smillie I, Yong K, Harris K, Wynne DM, Russell CJH. Socioeconomic influence on orofacial cleft patient care. Scott Med J. 2015;60(2):70–4. doi: 10.1177/0036933014564133 25504476

[22] Gamble C, Persson C, Willadsen E, Albery L, Soegaard Andersen H, Zattoni Antoneli M, et al. Timing of Primary Surgery for Cleft Palate. N Engl J Med. 2023;389(9):795–807. doi: 10.1056/NEJMoa2215162 37646677 PMC10880183

[23] Wehby GL, Castilla EE, Goco N, Rittler M, Cosentino V, Javois L, et al. The effect of systematic pediatric care on neonatal mortality and hospitalizations of infants born with oral clefts. BMC Pediatr. 2011;11:121. doi: 10.1186/1471-2431-11-121 22204448 PMC3277464

[24] Peck CJ, Gowda AU, Shultz BN, Wu RT, Bourdillon A, Singh A. The Effect of Surgical Timing on 30-Day Outcomes in Cleft Palate Repair. Plast Reconstr Surg. 2021;147(1):131–7. 33009328 10.1097/PRS.0000000000007458

