# Supplementary material for: The burden and epidemiology of orofacial clefts in Iran from 1990 to 2023: a Global Burden of Disease study with subnational socio-demographic index analysis
Source: PLoS One. 2026 Jul 8;21(7):e0352804. doi: 10.1371/journal.pone.0352804 (PMC13345398; doi:10.1371/journal.pone.0352804)
Supplement: Attachment — Submitted filename: RTR PLOS ONE-Orofacial cleft.doc [file pone.0352804.s011.doc]

**Response to Reviewers**:

**Manuscript reference number: PONE-D-26-17844**

**The burden and epidemiology of orofacial cleft and its socioeconomic associates in Iran from 1990 to 2023**

Dear Dr. Beiki,

Thank you for giving us the opportunity to revise our paper for potential publication in PLOS ONE. We are grateful to the reviewers and the Editors for their constructive and positive comments on our paper. We have addressed each of the comments in our revision, which has further improved the article. Below we detail our changes and respond to each of the comments in turn.

**Journal requirements**

When submitting your revision, we need you to address these additional requirements.

1.Please ensure that your manuscript meets PLOS ONE's style requirements, including those for file naming. The PLOS ONE style templates can be found at

<https://journals.plos.org/plosone/s/file?id=wjVg/PLOSOne_formatting_sample_main_body.pdf> and

<https://journals.plos.org/plosone/s/file?id=ba62/PLOSOne_formatting_sample_title_authors_affiliations.pdf>

2. PLOS requires an ORCID iD for the corresponding author in Editorial Manager on papers submitted after December 6th, 2016. Please ensure that you have an ORCID iD and that it is validated in Editorial Manager. To do this, go to ‘Update my Information’ (in the upper left-hand corner of the main menu), and click on the Fetch/Validate link next to the ORCID field. This will take you to the ORCID site and allow you to create a new iD or authenticate a pre-existing iD in Editorial Manager.

3. Please note that funding information should not appear in any section or other areas of your manuscript. We will only publish funding information present in the Funding Statement section of the online submission form. Please remove any funding-related text from the manuscript.

4. Please include captions for your Supporting Information files at the end of your manuscript, and update any in-text citations to match accordingly. Please see our Supporting Information guidelines for more information: <http://journals.plos.org/plosone/s/supporting-information>.

5. If the reviewer comments include a recommendation to cite specific previously published works, please review and evaluate these publications to determine whether they are relevant and should be cited. There is no requirement to cite these works unless the editor has indicated otherwise.

**Response:** Thank you for pointing this out. We implemented and considered the points.

**Reviewer Comments to Author:**

**Reviewer: 1**

**Comments to Author**

**Title**

The title appropriately identifies the topic and geographical scope but does not indicate that the study relies entirely on GBD-derived modeled estimates.

The phrase “socioeconomic associates” is vague and potentially misleading, as the analysis is limited to ecological SDI correlations rather than evaluation of socioeconomic determinants.

**Response:** Thank you very much for your valuable time and comments. To improve clarity and accurately reflect the scope and methodology of the study, we revised the title accordingly. It reads:

“The burden and epidemiology of orofacial clefts in Iran from 1990 to 2023: a Global Burden of Disease study with subnational socio-demographic index analysis”

**Abstract**

The abstract lacks sufficient methodological detail regarding how GBD estimates were obtained and analyzed.

Statements describing reductions as significant require clarification regarding statistical criteria.

The conclusion overstates policy implications related to maternal health programs despite no direct analysis of maternal risk factors.

**Response:** Accordingly, we revised the Methods section of the abstract to specify the GBD 2023 source, ICD-10 case definition, use of DisMod-MR 2.1 estimates, age-standardization, and uncertainty intervals. We also removed wording that could be interpreted as a formal significance claim and replaced it with neutral trend language. We moderated the conclusion to avoid direct causal implications about maternal health programs while still noting broader service-planning implications supported by the burden patterns observed.

**Introduction**

The rationale for conducting this study in Iran requires stronger justification beyond updating epidemiological estimates.

**Response:** We revised the introduction to emphasize that Iran has a large and geographically heterogeneous population, prior evidence has been mostly local or hospital-based, and no recent national and province-level time-trend assessment covering 1990–2023 had been available. We also clarified that the value of this study lies in informing subnational planning for cleft prevention, surgical access, and long-term rehabilitation.

The manuscript does not sufficiently explain the remaining knowledge gap considering multiple previous GBD-based OFC burden studies.

**Response:** We appreciate this important point. We clarified that although several GBD-based OFC studies have been published, they were primarily global or regional in scope and did not provide a long-term province-level assessment for Iran. We therefore strengthened the knowledge-gap statement to show that our study adds both temporal and subnational evidence for a country that has not been specifically characterized in recent GBD analyses.

The introduction lacks discussion regarding important distinctions between OFC subtypes and their epidemiologic implications.

**Response:** We revised the text to distinguish cleft lip with or without cleft palate from isolated cleft palate and to note that these subtypes differ in embryology, clinical course, treatment needs, and functional consequences. This helps justify why OFCs are an important congenital condition for epidemiologic monitoring, even when analyzed as a combined category in the GBD framework.

**Methods**

**Definition of Condition**

Combining all OFC phenotypes into a single category limits epidemiological interpretation because cleft palate and cleft lip with or without palate have different biological and clinical characteristics.

**Response:** We revised the Methods to make clear that the combined category was used because the GBD 2023 outputs for this analysis were available in that form, and we added a limitation stating that this approach does not allow subtype-specific inference.

The assumption of equal distribution among sequelae requires stronger justification because it may influence DALY estimation.

**Response:** We clarified that this was a simplifying assumption used within the GBD modeling framework when more granular sequela-specific data were unavailable. We also noted that this assumption may affect DALY estimates and should be considered when interpreting the results.

**Mathematical Modeling**

The manuscript reproduces GBD methodology but does not adequately clarify whether authors independently modeled estimates or extracted pre-generated outputs.

**Response:** We clarified that we did not independently re-estimate the GBD models. Instead, we extracted the finalized GBD 2023 outputs and used the published GBD framework to describe how those estimates were generated.

GBD assumptions are described extensively but their implications for interpretation in the Iranian setting are not discussed.

**Response:** We added text to emphasize that the results should be interpreted as ecological, population-level summaries rather than causal or individual-level socioeconomic effects.

**Statistical Analysis**

Details regarding smoothing spline models remain insufficient.

**Response:** We expanded the statistical analysis description to specify how the SDI relationship was assessed, what the models were used for, and how the smoothing was handled in R.

Model assumptions, smoothing parameters, and analytical procedures are not adequately described. The manuscript lacks reproducibility details including extraction parameters and analytical workflow.

**Response:** We added workflow details on extraction, organization, and analysis of the GBD outputs and clarified the software version used. It reads:

“For this analysis, SDI was treated as a continuous predictor and age-standardized OFC burden metrics were used as outcomes to explore potential non-linear patterns across provinces and over time. The smoothing spline curves were fitted in R using standard spline procedures, with the degree of smoothness selected automatically by the software to balance fit and parsimony. These analyses were descriptive rather than causal, and the observed SDI-burden patterns should be interpreted accordingly. The analytic workflow consisted of extracting the GBD outputs, restructuring them by location and year, calculating age-standardized rates and temporal changes, and then fitting the spline models.”

**Results**

Comparisons among Iran, MENA, and global estimates remain descriptive without formal comparative analyses.

**Response:** We agree that the comparisons across Iran, MENA, and global estimates are descriptive and were not based on formal hypothesis testing or pairwise comparative modeling. To avoid overinterpretation, we revised the text to present these findings as contextual comparisons rather than statistically tested differences.

Provincial variation is described extensively but remains observational and lacks spatial analytical methods.

**Response:** The provincial analysis in this study is descriptive and ecological in nature, based on GBD subnational estimates. We clarified this point in the Results and avoided language that might imply spatial clustering or spatial inference beyond the available data.

Age-related prevalence findings require cautious interpretation because prevalence in congenital conditions accumulates over time and may not reflect disease biology.

**Response:** We appreciate this important point. We revised the age-related section to avoid implying that prevalence patterns directly reflect disease occurrence at a given age. Instead, we clarified that prevalence in a congenital condition reflects accumulation over time, survival, and access to care, and therefore should be interpreted cautiously.

The higher DALY burden among females under five years is reported without exploration of possible explanations.

**Response:** We agree that this finding should be presented cautiously. Because the current analysis was descriptive, we did not attempt causal explanation in the Results. We revised the text to report the pattern without overinterpreting it and noted that possible explanations are better considered in the Discussion.

**Discussion**

Several interpretations attribute declining burden to improved surgical care, healthcare access, neonatal support, and health-system improvements despite these variables not being directly analyzed.

**Response:** We revised the Discussion to make clear that these factors are plausible explanations for the observed decline, but they were not directly measured in this study. We therefore changed the wording to present them as cautious interpretations rather than conclusions.

Explanations regarding referral patterns, healthcare inequalities, and reporting differences remain speculative.

**Response:** The revised text now identifies referral patterns, differential case ascertainment, and reporting completeness as possible contributors to provincial variation, while explicitly noting that these are hypotheses rather than directly tested explanations.

The discussion occasionally shifts from epidemiologic interpretation toward causal inference unsupported by presented data.

**Response:** We appreciate this important comment. We revised the Discussion to maintain an epidemiologic and descriptive focus throughout, removed causal language, and ensured that the interpretation remains aligned with the ecological and modeled nature of the data.

The markedly larger reduction in DALYs observed in Iran relative to global estimates warrants deeper discussion.

**Response:** We expanded the Discussion to compare Iran’s reduction with global and MENA trends more explicitly and to note that the larger decline may reflect a combination of improved survival, changes in disability patterns, and modeled estimation differences. We also clarified that this comparison should be interpreted cautiously because the estimates come from different geographic levels and modeling contexts. It reads:

“The larger decline in DALYs observed in Iran compared with global estimates (4) may reflect a combination of improving survival, changes in disability patterns, and differences in model-based estimation across geographic levels. However, this interpretation remains cautious because these factors were not directly tested in the present analysis (15).”

Ecological SDI findings should be interpreted cautiously to avoid ecological fallacy.

**Response:** As suggested, we revised the text to state clearly that the SDI analysis is ecological and descriptive, and that it cannot be interpreted as evidence of individual-level socioeconomic effects. It reads:

“This ecological association should be interpreted cautiously to avoid ecological fallacy, because province-level SDI does not necessarily represent the socioeconomic conditions of individual patients.”

**Limitations**

Important limitations are incompletely addressed.

**Response:** We agree and expanded the limitations substantially.

The study should explicitly discuss reliance on modeled rather than observed data.

**Response:** We added a direct statement that the analysis relies on GBD modeled estimates rather than primary observed registry data, and that this is an important limitation.

Limitations related to sparse provincial data, ecological analysis, inability to evaluate subtype-specific patterns, and inability to distinguish syndromic and non-syndromic OFCs should be acknowledged.

**Response:** We have added these limitations explicitly and noted that they constrain interpretation of provincial differences and prevent phenotype-specific and etiologic conclusions. It reads:

“Third, we could not evaluate the influence of specific risk factors at the provincial level within the GBD framework, so causal inferences about drivers of observed trends are limited. Fourth, because OFCs were analyzed as a combined category, we could not evaluate subtype-specific patterns for cleft lip with or without palate versus isolated cleft palate. Fifth, the GBD framework does not allow us to distinguish syndromic from non-syndromic OFCs, which limits etiologic interpretation. Sixth, the provincial and SDI analyses are ecological and should not be interpreted at the individual level. Nevertheless, the use of the most recent, internationally comparable GBD 2023 framework to produce a comprehensive, subnational picture of OFC burden for Iran across three decades provides useful context for understanding long-term trends and provincial inequalities. Future studies should compare GBD-derived estimates with Iranian congenital anomaly registry and hospital-based data when such data become available.”

**Conclusion**

The conclusion contains policy recommendations that extend beyond the presented analyses.

**Response:** We toned down the conclusion to focus more closely on the findings actually demonstrated in the study.

Statements regarding prevention strategies and healthcare interventions should be more closely aligned with observed epidemiological findings.

**Response:** We revised the conclusion to emphasize burden reduction, inequities, and the need for stronger surveillance and access to care, while avoiding overly specific preventive claims not directly supported by the analysis. It reads”

“Iran has experienced declines in OFC burden from 1990 to 2023, while national prevalence has remained relatively stable and provincial and age-specific disparities persist. The highest disability burden was among young children and was inversely associated with SDI, highlighting ongoing equity gaps in access to timely and comprehensive cleft care. These findings highlight persistent burden and inequity patterns that may inform future planning for surveillance, diagnosis, treatment access, and follow-up care. More specific prevention and health-system recommendations should be interpreted in light of future studies that directly examine risk factors, care pathways, and service availability.”

**Figures and Tables**

Figure 1 is visually crowded and difficult to interpret because of simultaneous presentation of multiple variables.

Figure 2 uses dual-axis presentation that complicates interpretation.

Figure 3 contains substantial visual overlap that limits readability.

Several figures emphasize descriptive visualization but provide limited analytical insight.

**Response:** We added further details in the figure legends to clarify that what each axis and color shows. The figures legends were provided in the Results section.

Table terminology such as “Pcs in rate” should be clearly defined.

**Response:** The abbreviation “Pcs in rate” (presumably “percentage change in rate”) was fully defined in the table footnote.

**References**

References are generally appropriate; however, the manuscript relies heavily on prior GBD-based publications, particularly References 4, 8, 10, 11, 12, and 13, which limits broader contextualization beyond burden-database analyses.

**Response:** We agree that the manuscript initially relied heavily on GBD-based sources and did not sufficiently broaden the contextual framing. We revised the Discussion and Introduction to include more context from Iranian epidemiological studies and other non-GBD literature, and we reduced overreliance on burden-database citations where broader clinical and public health context was more appropriate.

Greater discussion of how the present findings compare with and extend previous OFC burden studies, particularly References 4, 8, and 13, would strengthen the manuscript and better clarify its contribution.

**Response:** We expanded the Discussion to more explicitly compare our findings with prior global, regional, and national OFC burden studies. In particular, we now clarify that this study extends the existing literature by providing updated 1990–2023 estimates for Iran and by adding province-level interpretation, which had not been available in the earlier burden studies.

Additional comparison with Iranian epidemiological literature, including References 5, 6, 7, 14, 18, and 19, would improve interpretation and help contextualize similarities and discrepancies in prevalence estimates, geographic variation, and temporal trends.

**Response:** We have searched for the additional Iranian epidemiological literature into the manuscript, however there are few national estimates and most of them focused on the specific provinces or a city in a province. So, we tried to provide the comprehensive ones in the manuscript.

**Reviewer: 2**

**Comments to Author**

1. The manuscript mainly reproduces descriptive estimates from the GBD 2023 platform without providing substantial methodological novelty or additional analytical depth. Since the study relies entirely on secondary modeled data, the scientific contribution would be strengthened by including more original interpretation, validation with national registry data, or deeper exploration of factors potentially driving provincial disparities.

**Response:** Thank you very much for your time and your valuable comments. We agree that validation against national data sources would further strengthen interpretation of the findings. Although such validation was beyond the scope of the present manuscript, our research team is currently involved in separate projects evaluating Iranian hospital-based and registry-related congenital anomaly data, which may help contextualize future GBD-based estimates. We have revised the Introduction and Discussion to better emphasize the study’s contribution, namely the first comprehensive 1990–2023 national and subnational assessment of OFC burden in Iran using the latest GBD 2023 estimates. We also strengthened the limitations to acknowledge the absence of validation against national registry data and the descriptive nature of the provincial comparisons. The Introduction reads:

“Although prior studies reported the burden of OFCs at the global and regional levels using the burden of disease data (4, 8-10), important knowledge gaps remain for Iran. Most previous GBD-based analyses have been global or regional, and few have provided a long-term, province-level assessment for a specific country with marked geographic heterogeneity. In particular, no recent study has comprehensively described the OFC burden in Iran across both national and subnational levels over a 34-year period. Providing recent and updated data for health policymakers is important for decision making. In this study, we used the most recent global burden of disease (GBD) 2023 study to report the burden of OFCs in Iran and its provinces from 1990 to 2023.”

2. The interpretation of temporal trends appears somewhat overstated given the nature of GBD estimates. The reported declines in incidence, DALYs, and mortality may partly reflect changes in modeling methods, reporting completeness, survival, and healthcare access rather than true reductions in disease occurrence alone. The discussion should acknowledge this more carefully to avoid causal over-interpretation.

**Response:** We revised the Results and Discussion to use more cautious wording and to avoid presenting modeled changes as proof of causal reductions in disease occurrence. We now explicitly note that the observed trends may reflect a combination of true epidemiologic change, improved survival, changes in access to care, reporting completeness, and model-based estimation assumptions.

3. The manuscript repeatedly attributes provincial differences to factors such as prenatal screening, consanguinity, maternal exposures, or healthcare access; however, these variables were not directly analyzed in the study. While these explanations are plausible, they remain speculative within the current design and should be presented more cautiously unless supported by province-level evidence.

**Response:** We revised the Discussion to present these factors as possible explanations rather than conclusions. We also added clearer caution that the study is ecological and descriptive, and therefore cannot directly test provincial drivers such as screening patterns, consanguinity, maternal exposures, or service access. We highlighted it:

“In the present study, however, these possible determinants were not analyzed, so they should not be interpreted as causal explanations for the observed age- and sex-specific patterns.”

4. The use of SDI analysis is relevant, but the interpretation remains descriptive. The manuscript reports an inverse association between SDI and DALY burden, yet no quantitative assessment of correlation strength, model fit, or sensitivity analysis is provided beyond smoothing splines. Additional analytical detail would help readers better understand the robustness and public health significance of this association.

**Response:** We clarified that the smoothing spline analysis was intended to explore non-linear patterns rather than establish causal or individual-level relationships. We also strengthened the wording to avoid overinterpretation. Because the study is based on finalized GBD outputs, additional inferential modeling was not the primary aim; this is now stated more clearly in the text and acknowledged as a limitation.

5. The epidemiological interpretation of prevalence requires clarification. The manuscript reports stable prevalence despite marked declines in DALYs and mortality, but this important finding is only briefly discussed. Greater attention should be given to how improved survival, earlier surgical interventions, and long-term survivorship may contribute to persistent prevalence despite declining disability burden.

**Response:** The revised text now explains that stable prevalence in a congenital condition may reflect accumulation of surviving cases across age groups, improved survival, and longer-term survivorship after treatment, even when disability burden and mortality decline.

6. The study combines all orofacial cleft subtypes into a single category, which may mask important epidemiological and clinical differences between cleft lip, cleft palate, and combined cleft lip/palate. Since these subtypes differ in risk factors, severity, management, and outcomes, the absence of subtype-specific analyses limits the clinical interpretability of the findings.

**Response:**  We revised the Methods to clarify that OFCs were analyzed as a combined category because that was how the relevant GBD 2023 outputs were available for Iran and its provinces. We also added a limitation stating that this approach limits phenotype-specific interpretation and does not allow subtype-specific conclusions. It reads:

“Fourth, because OFCs were analyzed as a combined category, we could not evaluate subtype-specific patterns for cleft lip with or without palate versus isolated cleft palate.”

7. Several conclusions extend beyond what can be directly inferred from GBD data alone. Recommendations regarding maternal health programs, neonatal care expansion, and preventive strategies are reasonable, but the study itself does not evaluate intervention effectiveness or healthcare quality indicators. The conclusions should therefore remain more closely aligned with the descriptive nature of the analysis.

**Response:** We have revised the Conclusion to better match the descriptive scope of the study. The updated conclusion now focuses on burden patterns, inequities, and the need for continued surveillance and improved access to care, while avoiding stronger claims about intervention effectiveness that were not directly examined. It reads:

“Iran has experienced declines in OFC burden from 1990 to 2023, while national prevalence has remained relatively stable and provincial and age-specific disparities persist. The highest disability burden was among young children and was inversely associated with SDI, highlighting ongoing equity gaps in access to timely and comprehensive cleft care. These findings highlight persistent burden and inequity patterns that may inform future planning for surveillance, diagnosis, treatment access, and follow-up care. More specific prevention and health-system recommendations should be interpreted in light of future studies that directly examine risk factors, care pathways, and service availability.”

8. The manuscript would benefit from a more critical discussion of the limitations of GBD subnational estimates. Provincial-level estimates in settings with incomplete surveillance may carry substantial uncertainty, especially for congenital anomalies. Although uncertainty intervals are reported, the implications of sparse primary data and possible under-ascertainment should be discussed in greater depth.

**Response:** We appreciate this important point. We expanded the Limitations section to emphasize that the analysis relies on modeled GBD estimates, that provincial estimates may be affected by sparse primary data and under-ascertainment, and that the reported uncertainty intervals do not remove the need for cautious interpretation. We also clarified that ecological province-level findings should not be interpreted at the individual level. It reads:

“Our study has some limitations. First, the analysis relied on GBD modeled estimates rather than directly observed registry data, so the results depend on the assumptions and data inputs used in the GBD framework. Although this approach enables standardized comparisons across time and location, it may not fully capture local clinical reality. Second, the GBD case definition and the need to distribute sequelae proportions in the absence of granular local data require simplifying assumptions, which may not perfectly reflect Iran’s clinical reality. Third, we could not evaluate the influence of specific risk factors at the provincial level within the GBD framework, so causal inferences about drivers of observed trends are limited. Fourth, because OFCs were analyzed as a combined category, we could not evaluate subtype-specific patterns for cleft lip with or without palate versus isolated cleft palate. Fifth, the GBD framework does not allow us to distinguish syndromic from non-syndromic OFCs, which limits etiologic interpretation. Sixth, the provincial and SDI analyses are ecological and should not be interpreted at the individual level. Nevertheless, the use of the most recent, internationally comparable GBD 2023 framework to produce a comprehensive, subnational picture of OFC burden for Iran across three decades provides useful context for understanding long-term trends and provincial inequalities. Future studies should compare GBD-derived estimates with Iranian congenital anomaly registry and hospital-based data when such data become available.”
