# Supplementary material for: The burden and epidemiology of orofacial clefts in Iran from 1990 to 2023: a Global Burden of Disease study with subnational socio-demographic index analysis
Source: PLoS One. 2026 Jul 8;21(7):e0352804. doi: 10.1371/journal.pone.0352804 (PMC13345398; doi:10.1371/journal.pone.0352804)
Supplement: Attachment — Submitted filename: R2 RTR PLOS ONE-Orofacial cleft.doc [file pone.0352804.s012.doc]

**Response to Reviewers**:

**Manuscript reference number: PONE-D-26-17844R1**

**The burden and epidemiology of orofacial clefts in Iran from 1990 to 2023: a Global Burden of Disease study with subnational socio-demographic index analysis**

Dear Dr. Beiki,

Thank you for giving us the opportunity to revise our paper for potential publication in PLOS ONE. We are grateful to the reviewers and the Editors for their constructive and positive comments on our paper. We have addressed each of the comments in our revision, which has further improved the article. Below we detail our changes and respond to each of the comments in turn.

**Reviewer Comments to Author:**

**Reviewer: 1**

**Comments to Author**

The authors have addressed my comments satisfactorily and significantly improved the manuscript. The revised version is clearer, more balanced, and methodologically transparent.

**Response:** Thank you very much for reviewing the revised manuscript and your positive feedback.

**Reviewer: 2**

1. The manuscript should clearly distinguish whether the authors conducted any independent modeling or simply analyzed pre-existing IHME/GBD outputs. As currently written, this distinction is blurred. It needs to explicitly state that no new primary DisMod-MR 2.1 modeling was performed, and that the work is based on descriptive analysis of extracted GBD data rather than reproduction of the original modeling framework.

**Response:** Thank you so much for your time to review the revised manuscript and your valuable comments. We have revised the Methods to state that this study is based on a descriptive secondary analysis of finalized GBD 2023 outputs and that no new primary DisMod-MR 2.1 modeling was performed by the authors. We also clarified that the manuscript does not reproduce the original IHME modeling process but instead summarizes and interprets the available estimates for Iran and its provinces. It reads:

“We extracted the finalized GBD 2023 estimates and conducted a descriptive secondary analysis of these pre-generated outputs. No new primary DisMod-MR 2.1 modeling was performed in this study.”

“We did not independently re-fit the full GBD model. Instead, we extracted and analyzed the finalized GBD 2023 estimates as a descriptive secondary analysis.”

2. The aggregation of all orofacial clefts (ICD-10 Q35–Q37) into a single category conceals meaningful clinical and biological differences between subtypes such as cleft palate and cleft lip with or without cleft palate. While this grouping may be constrained by the structure of available GBD data, the manuscript should acknowledge that it reduces clinical granularity and may limit the interpretability and real-world applicability of burden estimates.

**Response:** We appreciate this point and agree that combining all OFC phenotypes into a single category reduces clinical granularity. We revised the manuscript to explain that this aggregation was necessary because the relevant GBD 2023 outputs were available only in combined form for Iran and its provinces. We also added text noting that this approach improves comparability across locations and years but limits phenotype-specific interpretation and real-world clinical applicability. It reads:

“Although OFC phenotypes differ in biological and clinical characteristics, they were analyzed as a combined category because this is how the GBD 2023 outputs were available for Iran and its provinces. This grouping improves comparability across years and locations, but it reduces clinical granularity and limits phenotype-specific interpretation and real-world applicability of the burden estimates. The equal-distribution assumption among sequelae was used within the GBD framework when more granular sequela-specific data were unavailable, and we acknowledge that this simplifying assumption may influence DALY estimation.”

3. The use of smoothing splines to model the relationship between SDI and burden metrics is not sufficiently described. Key details such as smoothing parameters, degrees of freedom, and model fit assessment are missing. The analysis should be explicitly framed as an exploratory, descriptive approach rather than inferential modeling, with clearer justification of how curve smoothness was selected to avoid the impression of arbitrary fitting.

**Response:** We appreciate your suggestion and have expanded the description of the SDI analysis in the Methods. We emphasize that the smoothing spline analysis was intended as an exploratory visualization of the relationship between SDI and age-standardized OFC burden metrics and was not designed as an inferential or predictive model. The spline curves were fitted using standard smoothing spline procedures in R, with the smoothing parameter selected automatically through generalized cross-validation, which balances model fit against complexity and helps avoid overfitting. We did not pre-specify the degrees of freedom or manually tune the smoothness parameter, as the objective was descriptive visualization rather than formal hypothesis testing or model optimization. Consequently, we did not perform additional assessments of model fit or sensitivity analyses, and the resulting curves should be interpreted as graphical summaries of non-linear ecological patterns rather than evidence of causal or statistically significant associations. It reads:

“Spline curves were fitted in R using standard smoothing spline procedures, with the smoothing parameter selected automatically by generalized cross-validation, allowing the algorithm to balance goodness of fit against model complexity and reduce the risk of overfitting. No degrees of freedom or smoothing parameters were manually specified or optimized, because the analysis was intended to provide descriptive visualization rather than formal statistical inference or prediction. Accordingly, no hypothesis testing or model-based causal interpretation was performed, and the observed SDI–burden relationships should be interpreted as population-level ecological patterns rather than individual-level associations.”

4. The observed inverse association between provincial SDI and DALYs is interpreted too strongly in places, risking ecological fallacy. Because the data are aggregated at the provincial level, these findings should not be extended to individual-level socioeconomic risk. The results should be framed strictly as area-level patterns rather than implying individual-level relationships.
**Response:** We revised the Results and Discussion to make clear that the inverse association between provincial SDI and DALY burden is an ecological, province-level pattern and should not be interpreted as evidence of individual-level socioeconomic risk. We also noted the risk of ecological fallacy and avoided wording that could imply causality at the individual level. It reads:

“The burden of OFCs, represented by the age-standardized DALY rate per 100,000 population, showed an inverse area-level association with the SDI.”

“Because these findings are based on ecological, modeled estimates, they describe population-level patterns rather than individual-level associations.”

“This ecological association should be interpreted cautiously to avoid ecological fallacy, because province-level SDI does not necessarily represent the socioeconomic conditions of individual patients.”

5. The manuscript highlights stable prevalence over time alongside a substantial reduction in DALYs and mortality, but this pattern is not well explained. A clearer interpretation is needed, likely involving improved survival, better long-term management, and the accumulation of treated individuals who continue to live with the condition, which maintains prevalence even as severity and mortality decline.

**Response:** We expanded the Discussion to better explain why prevalence may remain stable even when DALYs and mortality decline. The revised text now notes that in a congenital condition such as OFC, prevalence may accumulate across age groups because treated individuals survive longer, while disability burden and mortality may decrease with improved survival, earlier management, and better long-term care. This interpretation is now stated more clearly and cautiously. It reads:

“The decline in DALYs and deaths is consistent with a broader improvement in population health and may reflect improved survival, better long-term management, and reduced severity among affected individuals over time, but our study cannot determine the exact reason (22). In particular, in a congenital condition such as OFC, prevalence may remain stable because treated individuals continue to survive into later age groups, even as mortality and disability burden decline. Moreover, population health gains such as reductions in infant mortality and improved neonatal services can reduce OFC-related morbidity (23). Similarly, observed provincial inequalities may reflect differences in access, referral, ascertainment, and service availability, but the present analysis does not allow these mechanisms to be distinguished (18, 19). Accordingly, these factors should be considered plausible hypotheses rather than confirmed determinants of the observed trends.”

6. Several causal explanations for declining DALYs and mortality are stated too definitively. Since the GBD dataset does not directly measure healthcare access, prenatal screening, or underlying risk factors, these should be reframed as possible contributing hypotheses rather than confirmed drivers of change.

**Response:** We revised the Discussion to frame surgical advances, neonatal care, healthcare access, and system improvements as possible contributing hypotheses rather than confirmed drivers of change. We also added explicit caution that these factors were not directly measured in the present analysis, so causal inference is not possible from the current dataset. It reads:

“The larger decline in DALYs observed in Iran compared with global estimates (4) might be partially due to a combination of improving survival, changes in disability patterns, and differences in model-based estimation across geographic levels. However, this interpretation remains cautious because these factors were not directly tested in the present analysis. Also, these should be considered as possible contributing hypotheses rather than confirmed drivers of change (15).”

7. The policy recommendations extend beyond what can be justified by a descriptive epidemiological analysis. Specific clinical or preventive interventions should be avoided. Instead, conclusions should focus on higher-level public health implications such as strengthening congenital anomaly surveillance systems, improving registry completeness, and supporting broad resource allocation and health system planning.

**Response:** Thank you for your recommendations. We revised the conclusion to focus on the observed burden patterns, provincial disparities, and the implications for surveillance, registry completeness, resource allocation, and health-system planning. We removed or softened recommendations that went beyond what can be directly supported by the GBD data. It reads:

“Iran has experienced declines in OFC burden from 1990 to 2023, while national prevalence has remained relatively stable and provincial and age-specific disparities persist. The highest disability burden was among young children and was inversely associated with SDI, highlighting ongoing equity gaps in access to timely and comprehensive cleft care. These findings highlight persistent burden and inequity patterns that may inform future planning for surveillance, registry completeness, diagnosis, treatment access, and follow-up care. More specific prevention and health-system recommendations should be interpreted in light of future studies that directly examine risk factors, care pathways, and service availability.”
