# Supplementary material for: The burden and epidemiology of orofacial clefts in Iran from 1990 to 2023: a Global Burden of Disease study with subnational socio-demographic index analysis
Source: PLoS One. 2026 Jul 8;21(7):e0352804. doi: 10.1371/journal.pone.0352804 (PMC13345398; doi:10.1371/journal.pone.0352804)
Supplement: Attachment — Submitted filename: reviewer comments.docx [file pone.0352804.s009.docx]

**The burden and epidemiology of orofacial cleft and its socioeconomic associates in Iran from 1990 to 2023**

**Title**

The title appropriately identifies the topic and geographical scope but does not indicate that the study relies entirely on GBD-derived modeled estimates.

The phrase “socioeconomic associates” is vague and potentially misleading, as the analysis is limited to ecological SDI correlations rather than evaluation of socioeconomic determinants.

**Abstract**

The abstract lacks sufficient methodological detail regarding how GBD estimates were obtained and analyzed.

Statements describing reductions as significant require clarification regarding statistical criteria.

The conclusion overstates policy implications related to maternal health programs despite no direct analysis of maternal risk factors.

**Introduction**

The rationale for conducting this study in Iran requires stronger justification beyond updating epidemiological estimates.

The manuscript does not sufficiently explain the remaining knowledge gap considering multiple previous GBD-based OFC burden studies.

The introduction lacks discussion regarding important distinctions between OFC subtypes and their epidemiologic implications.

**Methods**

**Definition of Condition**

Combining all OFC phenotypes into a single category limits epidemiological interpretation because cleft palate and cleft lip with or without palate have different biological and clinical characteristics.

The assumption of equal distribution among sequelae requires stronger justification because it may influence DALY estimation.

**Mathematical Modeling**

The manuscript reproduces GBD methodology but does not adequately clarify whether authors independently modeled estimates or extracted pre-generated outputs.

GBD assumptions are described extensively but their implications for interpretation in the Iranian setting are not discussed.

**Statistical Analysis**

Details regarding smoothing spline models remain insufficient.

Model assumptions, smoothing parameters, and analytical procedures are not adequately described.

The manuscript lacks reproducibility details including extraction parameters and analytical workflow.

**Results**

Comparisons among Iran, MENA, and global estimates remain descriptive without formal comparative analyses.

Provincial variation is described extensively but remains observational and lacks spatial analytical methods.

Age-related prevalence findings require cautious interpretation because prevalence in congenital conditions accumulates over time and may not reflect disease biology.

The higher DALY burden among females under five years is reported without exploration of possible explanations.

**Discussion**

Several interpretations attribute declining burden to improved surgical care, healthcare access, neonatal support, and health-system improvements despite these variables not being directly analyzed.

Explanations regarding referral patterns, healthcare inequalities, and reporting differences remain speculative.

The discussion occasionally shifts from epidemiologic interpretation toward causal inference unsupported by presented data.

The markedly larger reduction in DALYs observed in Iran relative to global estimates warrants deeper discussion.

Ecological SDI findings should be interpreted cautiously to avoid ecological fallacy.

**Limitations**

Important limitations are incompletely addressed.

The study should explicitly discuss reliance on modeled rather than observed data.

Limitations related to sparse provincial data, ecological analysis, inability to evaluate subtype-specific patterns, and inability to distinguish syndromic and non-syndromic OFCs should be acknowledged.

**Conclusion**

The conclusion contains policy recommendations that extend beyond the presented analyses.

Statements regarding prevention strategies and healthcare interventions should be more closely aligned with observed epidemiological findings.

**Figures and Tables**

Figure 1 is visually crowded and difficult to interpret because of simultaneous presentation of multiple variables.

Figure 2 uses dual-axis presentation that complicates interpretation.

Figure 3 contains substantial visual overlap that limits readability.

Several figures emphasize descriptive visualization but provide limited analytical insight.

Table terminology such as “Pcs in rate” should be clearly defined.

**References**

References are generally appropriate; however, the manuscript relies heavily on prior GBD-based publications, particularly References 4, 8, 10, 11, 12, and 13, which limits broader contextualization beyond burden-database analyses.

Greater discussion of how the present findings compare with and extend previous OFC burden studies, particularly References 4, 8, and 13, would strengthen the manuscript and better clarify its contribution.

Additional comparison with Iranian epidemiological literature, including References 5, 6, 7, 14, 18, and 19, would improve interpretation and help contextualize similarities and discrepancies in prevalence estimates, geographic variation, and temporal trends.
