# Supplementary material for: The burden and epidemiology of orofacial clefts in Iran from 1990 to 2023: a Global Burden of Disease study with subnational socio-demographic index analysis
Source: PLoS One. 2026 Jul 8;21(7):e0352804. doi: 10.1371/journal.pone.0352804 (PMC13345398; doi:10.1371/journal.pone.0352804)
Supplement: Peer review history

# Peer review: Decision Letter 0

**DOI:** 10.1371/journal.pone.0352804.r001

**Authors:** Omid Beiki

17 May 2026

PONE-D-26-17844

The burden and epidemiology of orofacial cleft and its socioeconomic associates in Iran from 1990 to 2023

PLOS One

Dear Dr. Bakhtiari,

Thank you for submitting your manuscript to PLOS ONE. After careful consideration, we feel that it has merit but does not fully meet PLOS ONE’s publication criteria as it currently stands. Therefore, we invite you to submit a revised version of the manuscript that addresses the points raised during the review process.

Please submit your revised manuscript by Jul 01 2026 11:59PM. If you will need more time than this to complete your revisions, please reply to this message or contact the journal office at plosone@plos.org. When you're ready to submit your revision, log on to https://www.editorialmanager.com/pone/ and select the 'Submissions Needing Revision' folder to locate your manuscript file.

Please include the following items when submitting your revised manuscript:

- A letter that responds to each point raised by the academic editor and reviewer(s). You should upload this letter as a separate file labeled 'Response to Reviewers'.
- A marked-up copy of your manuscript that highlights changes made to the original version. You should upload this as a separate file labeled 'Revised Manuscript with Track Changes'.
- An unmarked version of your revised paper without tracked changes. You should upload this as a separate file labeled 'Manuscript'.

If you would like to make changes to your financial disclosure, please include your updated statement in your cover letter. Guidelines for resubmitting your figure files are available below the reviewer comments at the end of this letter.

If applicable, we recommend that you deposit your laboratory protocols in protocols.io to enhance the reproducibility of your results. Protocols.io assigns your protocol its own identifier (DOI) so that it can be cited independently in the future. For instructions see: https://journals.plos.org/plosone/s/submission-guidelines#loc-laboratory-protocols. Additionally, PLOS ONE offers an option for publishing peer-reviewed Lab Protocol articles, which describe protocols hosted on protocols.io. Read more information on sharing protocols at https://plos.org/protocols?utm_medium=editorial-email&utm_source=authorletters&utm_campaign=protocols.

As the corresponding author, your ORCID iD is verified in the submission system and will appear in the published article. PLOS supports the use of ORCID, and we encourage all coauthors to register for an ORCID iD and use it as well. Please encourage your coauthors to verify their ORCID iD within the submission system before final acceptance, as unverified ORCID iDs will not appear in the published article. *Only*  the individual author can complete the verification step; PLOS staff *cannot*  verify ORCID iDs on behalf of authors.

We look forward to receiving your revised manuscript.

Kind regards,

Omid Beiki, M.D., Ph.D.

Academic Editor

PLOS One

Journal Requirements:

When submitting your revision, we need you to address these additional requirements.

1.Please ensure that your manuscript meets PLOS ONE's style requirements, including those for file naming. The PLOS ONE style templates can be found at

https://journals.plos.org/plosone/s/file?id=wjVg/PLOSOne_formatting_sample_main_body.pdf and

https://journals.plos.org/plosone/s/file?id=ba62/PLOSOne_formatting_sample_title_authors_affiliations.pdf

2. PLOS requires an ORCID iD for the corresponding author in Editorial Manager on papers submitted after December 6th, 2016. Please ensure that you have an ORCID iD and that it is validated in Editorial Manager. To do this, go to ‘Update my Information’ (in the upper left-hand corner of the main menu), and click on the Fetch/Validate link next to the ORCID field. This will take you to the ORCID site and allow you to create a new iD or authenticate a pre-existing iD in Editorial Manager.

3. Please note that funding information should not appear in any section or other areas of your manuscript. We will only publish funding information present in the Funding Statement section of the online submission form. Please remove any funding-related text from the manuscript.

4. Please include captions for your Supporting Information files at the end of your manuscript, and update any in-text citations to match accordingly. Please see our Supporting Information guidelines for more information: http://journals.plos.org/plosone/s/supporting-information.

5. If the reviewer comments include a recommendation to cite specific previously published works, please review and evaluate these publications to determine whether they are relevant and should be cited. There is no requirement to cite these works unless the editor has indicated otherwise.

[Note: HTML markup is below. Please do not edit.]

Reviewer's Responses to Questions

**Comments to the Author**

1. Is the manuscript technically sound, and do the data support the conclusions?

The manuscript must describe a technically sound piece of scientific research with data that supports the conclusions. Experiments must have been conducted rigorously, with appropriate controls, replication, and sample sizes. The conclusions must be drawn appropriately based on the data presented.

Reviewer #1: Partly

Reviewer #2: Yes

**********

2. Has the statistical analysis been performed appropriately and rigorously?

Reviewer #1: No

Reviewer #2: Yes

**********

3. Have the authors made all data underlying the findings in their manuscript fully available?

The PLOS Data policy requires authors to make all data underlying the findings described in their manuscript fully available without restriction, with rare exception (please refer to the Data Availability Statement in the manuscript PDF file). The data should be provided as part of the manuscript or its supporting information, or deposited to a public repository. For example, in addition to summary statistics, the data points behind means, medians and variance measures should be available. If there are restrictions on publicly sharing data—e.g. participant privacy or use of data from a third party—those must be specified.

Reviewer #1: Yes

Reviewer #2: Yes

**********

4. Is the manuscript presented in an intelligible fashion and written in standard English?

PLOS ONE does not copyedit accepted manuscripts, so the language in submitted articles must be clear, correct, and unambiguous. Any typographical or grammatical errors should be corrected at revision, so please note any specific errors here.

Reviewer #1: Yes

Reviewer #2: Yes

**********

5. Review Comments to the Author

Please use the space provided to explain your answers to the questions above. You may also include additional comments for the author, including concerns about dual publication, research ethics, or publication ethics. (Please upload your review as an attachment if it exceeds 20,000 characters)

Reviewer #1: Title

The title appropriately identifies the topic and geographical scope but does not indicate that the study relies entirely on GBD-derived modeled estimates.

The phrase “socioeconomic associates” is vague and potentially misleading, as the analysis is limited to ecological SDI correlations rather than evaluation of socioeconomic determinants.

Abstract

The abstract lacks sufficient methodological detail regarding how GBD estimates were obtained and analyzed.

Statements describing reductions as significant require clarification regarding statistical criteria.

The conclusion overstates policy implications related to maternal health programs despite no direct analysis of maternal risk factors.

Introduction

The rationale for conducting this study in Iran requires stronger justification beyond updating epidemiological estimates.

The manuscript does not sufficiently explain the remaining knowledge gap considering multiple previous GBD-based OFC burden studies.

The introduction lacks discussion regarding important distinctions between OFC subtypes and their epidemiologic implications.

Methods

Definition of Condition

Combining all OFC phenotypes into a single category limits epidemiological interpretation because cleft palate and cleft lip with or without palate have different biological and clinical characteristics.

The assumption of equal distribution among sequelae requires stronger justification because it may influence DALY estimation.

Mathematical Modeling

The manuscript reproduces GBD methodology but does not adequately clarify whether authors independently modeled estimates or extracted pre-generated outputs.

GBD assumptions are described extensively but their implications for interpretation in the Iranian setting are not discussed.

Statistical Analysis

Details regarding smoothing spline models remain insufficient.

Model assumptions, smoothing parameters, and analytical procedures are not adequately described.

The manuscript lacks reproducibility details including extraction parameters and analytical workflow.

Results

Comparisons among Iran, MENA, and global estimates remain descriptive without formal comparative analyses.

Provincial variation is described extensively but remains observational and lacks spatial analytical methods.

Age-related prevalence findings require cautious interpretation because prevalence in congenital conditions accumulates over time and may not reflect disease biology.

The higher DALY burden among females under five years is reported without exploration of possible explanations.

Discussion

Several interpretations attribute declining burden to improved surgical care, healthcare access, neonatal support, and health-system improvements despite these variables not being directly analyzed.

Explanations regarding referral patterns, healthcare inequalities, and reporting differences remain speculative.

The discussion occasionally shifts from epidemiologic interpretation toward causal inference unsupported by presented data.

The markedly larger reduction in DALYs observed in Iran relative to global estimates warrants deeper discussion.

Ecological SDI findings should be interpreted cautiously to avoid ecological fallacy.

Limitations

Important limitations are incompletely addressed.

The study should explicitly discuss reliance on modeled rather than observed data.

Limitations related to sparse provincial data, ecological analysis, inability to evaluate subtype-specific patterns, and inability to distinguish syndromic and non-syndromic OFCs should be acknowledged.

Conclusion

The conclusion contains policy recommendations that extend beyond the presented analyses.

Statements regarding prevention strategies and healthcare interventions should be more closely aligned with observed epidemiological findings.

Figures and Tables

Figure 1 is visually crowded and difficult to interpret because of simultaneous presentation of multiple variables.

Figure 2 uses dual-axis presentation that complicates interpretation.

Figure 3 contains substantial visual overlap that limits readability.

Several figures emphasize descriptive visualization but provide limited analytical insight.

Table terminology such as “Pcs in rate” should be clearly defined.

References

References are generally appropriate; however, the manuscript relies heavily on prior GBD-based publications, particularly References 4, 8, 10, 11, 12, and 13, which limits broader contextualization beyond burden-database analyses.

Greater discussion of how the present findings compare with and extend previous OFC burden studies, particularly References 4, 8, and 13, would strengthen the manuscript and better clarify its contribution.

Additional comparison with Iranian epidemiological literature, including References 5, 6, 7, 14, 18, and 19, would improve interpretation and help contextualize similarities and discrepancies in prevalence estimates, geographic variation, and temporal trends.

Reviewer #2: 1. The manuscript mainly reproduces descriptive estimates from the GBD 2023 platform without providing substantial methodological novelty or additional analytical depth. Since the study relies entirely on secondary modeled data, the scientific contribution would be strengthened by including more original interpretation, validation with national registry data, or deeper exploration of factors potentially driving provincial disparities.

2. The interpretation of temporal trends appears somewhat overstated given the nature of GBD estimates. The reported declines in incidence, DALYs, and mortality may partly reflect changes in modeling methods, reporting completeness, survival, and healthcare access rather than true reductions in disease occurrence alone. The discussion should acknowledge this more carefully to avoid causal over-interpretation.

3. The manuscript repeatedly attributes provincial differences to factors such as prenatal screening, consanguinity, maternal exposures, or healthcare access; however, these variables were not directly analyzed in the study. While these explanations are plausible, they remain speculative within the current design and should be presented more cautiously unless supported by province-level evidence.

4. The use of SDI analysis is relevant, but the interpretation remains descriptive. The manuscript reports an inverse association between SDI and DALY burden, yet no quantitative assessment of correlation strength, model fit, or sensitivity analysis is provided beyond smoothing splines. Additional analytical detail would help readers better understand the robustness and public health significance of this association.

5. The epidemiological interpretation of prevalence requires clarification. The manuscript reports stable prevalence despite marked declines in DALYs and mortality, but this important finding is only briefly discussed. Greater attention should be given to how improved survival, earlier surgical interventions, and long-term survivorship may contribute to persistent prevalence despite declining disability burden.

6. The study combines all orofacial cleft subtypes into a single category, which may mask important epidemiological and clinical differences between cleft lip, cleft palate, and combined cleft lip/palate. Since these subtypes differ in risk factors, severity, management, and outcomes, the absence of subtype-specific analyses limits the clinical interpretability of the findings.

7. Several conclusions extend beyond what can be directly inferred from GBD data alone. Recommendations regarding maternal health programs, neonatal care expansion, and preventive strategies are reasonable, but the study itself does not evaluate intervention effectiveness or healthcare quality indicators. The conclusions should therefore remain more closely aligned with the descriptive nature of the analysis.

8. The manuscript would benefit from a more critical discussion of the limitations of GBD subnational estimates. Provincial-level estimates in settings with incomplete surveillance may carry substantial uncertainty, especially for congenital anomalies. Although uncertainty intervals are reported, the implications of sparse primary data and possible under-ascertainment should be discussed in greater depth.

**********

6. PLOS authors have the option to publish the peer review history of their article (what does this mean?). If published, this will include your full peer review and any attached files.

If you choose “no”, your identity will remain anonymous but your review may still be made public.

**Do you want your identity to be public for this peer review?**  For information about this choice, including consent withdrawal, please see our Privacy Policy.

Reviewer #1: No

Reviewer #2: No

**********

[NOTE: If reviewer comments were submitted as an attachment file, they will be attached to this email and accessible via the submission site. Please log into your account, locate the manuscript record, and check for the action link "View Attachments". If this link does not appear, there are no attachment files.]

To ensure your figures meet our technical requirements, please review our figure guidelines: https://journals.plos.org/plosone/s/figures

You may also use PLOS’s free figure tool, NAAS, to help you prepare publication quality figures: https://journals.plos.org/plosone/s/figures#loc-tools-for-figure-preparation.

NAAS will assess whether your figures meet our technical requirements by comparing each figure against our figure specifications.

Attachment Submitted filename: reviewer comments.docx

# Author comment: Author response to Decision Letter 1

**DOI:** 10.1371/journal.pone.0352804.r002

24 May 2026

Response to Reviewers:

Manuscript reference number: PONE-D-26-17844

The burden and epidemiology of orofacial cleft and its socioeconomic associates in Iran from 1990 to 2023

Dear Dr. Beiki,

Thank you for giving us the opportunity to revise our paper for potential publication in PLOS ONE. We are grateful to the reviewers and the Editors for their constructive and positive comments on our paper. We have addressed each of the comments in our revision, which has further improved the article. Below we detail our changes and respond to each of the comments in turn.

Journal requirements

When submitting your revision, we need you to address these additional requirements.

1.Please ensure that your manuscript meets PLOS ONE's style requirements, including those for file naming. The PLOS ONE style templates can be found at

https://journals.plos.org/plosone/s/file?id=wjVg/PLOSOne_formatting_sample_main_body.pdf and

https://journals.plos.org/plosone/s/file?id=ba62/PLOSOne_formatting_sample_title_authors_affiliations.pdf

2. PLOS requires an ORCID iD for the corresponding author in Editorial Manager on papers submitted after December 6th, 2016. Please ensure that you have an ORCID iD and that it is validated in Editorial Manager. To do this, go to ‘Update my Information’ (in the upper left-hand corner of the main menu), and click on the Fetch/Validate link next to the ORCID field. This will take you to the ORCID site and allow you to create a new iD or authenticate a pre-existing iD in Editorial Manager.

3. Please note that funding information should not appear in any section or other areas of your manuscript. We will only publish funding information present in the Funding Statement section of the online submission form. Please remove any funding-related text from the manuscript.

4. Please include captions for your Supporting Information files at the end of your manuscript, and update any in-text citations to match accordingly. Please see our Supporting Information guidelines for more information: http://journals.plos.org/plosone/s/supporting-information.

5. If the reviewer comments include a recommendation to cite specific previously published works, please review and evaluate these publications to determine whether they are relevant and should be cited. There is no requirement to cite these works unless the editor has indicated otherwise.

Response: Thank you for pointing this out. We implemented and considered the points.

Reviewer Comments to Author:

Reviewer: 1

Comments to Author

Title

The title appropriately identifies the topic and geographical scope but does not indicate that the study relies entirely on GBD-derived modeled estimates.

The phrase “socioeconomic associates” is vague and potentially misleading, as the analysis is limited to ecological SDI correlations rather than evaluation of socioeconomic determinants.

Response: Thank you very much for your valuable time and comments. To improve clarity and accurately reflect the scope and methodology of the study, we revised the title accordingly. It reads:

“The burden and epidemiology of orofacial clefts in Iran from 1990 to 2023: a Global Burden of Disease study with subnational socio-demographic index analysis”

Abstract

The abstract lacks sufficient methodological detail regarding how GBD estimates were obtained and analyzed.

Statements describing reductions as significant require clarification regarding statistical criteria.

The conclusion overstates policy implications related to maternal health programs despite no direct analysis of maternal risk factors.

Response: Accordingly, we revised the Methods section of the abstract to specify the GBD 2023 source, ICD-10 case definition, use of DisMod-MR 2.1 estimates, age-standardization, and uncertainty intervals. We also removed wording that could be interpreted as a formal significance claim and replaced it with neutral trend language. We moderated the conclusion to avoid direct causal implications about maternal health programs while still noting broader service-planning implications supported by the burden patterns observed.

Introduction

The rationale for conducting this study in Iran requires stronger justification beyond updating epidemiological estimates.

Response: We revised the introduction to emphasize that Iran has a large and geographically heterogeneous population, prior evidence has been mostly local or hospital-based, and no recent national and province-level time-trend assessment covering 1990–2023 had been available. We also clarified that the value of this study lies in informing subnational planning for cleft prevention, surgical access, and long-term rehabilitation.

The manuscript does not sufficiently explain the remaining knowledge gap considering multiple previous GBD-based OFC burden studies.

Response: We appreciate this important point. We clarified that although several GBD-based OFC studies have been published, they were primarily global or regional in scope and did not provide a long-term province-level assessment for Iran. We therefore strengthened the knowledge-gap statement to show that our study adds both temporal and subnational evidence for a country that has not been specifically characterized in recent GBD analyses.

The introduction lacks discussion regarding important distinctions between OFC subtypes and their epidemiologic implications.

Response: We revised the text to distinguish cleft lip with or without cleft palate from isolated cleft palate and to note that these subtypes differ in embryology, clinical course, treatment needs, and functional consequences. This helps justify why OFCs are an important congenital condition for epidemiologic monitoring, even when analyzed as a combined category in the GBD framework.

Methods

Definition of Condition

Combining all OFC phenotypes into a single category limits epidemiological interpretation because cleft palate and cleft lip with or without palate have different biological and clinical characteristics.

Response: We revised the Methods to make clear that the combined category was used because the GBD 2023 outputs for this analysis were available in that form, and we added a limitation stating that this approach does not allow subtype-specific inference.

The assumption of equal distribution among sequelae requires stronger justification because it may influence DALY estimation.

Response: We clarified that this was a simplifying assumption used within the GBD modeling framework when more granular sequela-specific data were unavailable. We also noted that this assumption may affect DALY estimates and should be considered when interpreting the results.

Mathematical Modeling

The manuscript reproduces GBD methodology but does not adequately clarify whether authors independently modeled estimates or extracted pre-generated outputs.

Response: We clarified that we did not independently re-estimate the GBD models. Instead, we extracted the finalized GBD 2023 outputs and used the published GBD framework to describe how those estimates were generated.

GBD assumptions are described extensively but their implications for interpretation in the Iranian setting are not discussed.

Response: We added text to emphasize that the results should be interpreted as ecological, population-level summaries rather than causal or individual-level socioeconomic effects.

Statistical Analysis

Details regarding smoothing spline models remain insufficient.

Response: We expanded the statistical analysis description to specify how the SDI relationship was assessed, what the models were used for, and how the smoothing was handled in R.

Model assumptions, smoothing parameters, and analytical procedures are not adequately described. The manuscript lacks reproducibility details including extraction parameters and analytical workflow.

Response: We added workflow details on extraction, organization, and analysis of the GBD outputs and clarified the software version used. It reads:

“For this analysis, SDI was treated as a continuous predictor and age-standardized OFC burden metrics were used as outcomes to explore potential non-linear patterns across provinces and over time. The smoothing spline curves were fitted in R using standard spline procedures, with the degree of smoothness selected automatically by the software to balance fit and parsimony. These analyses were descriptive rather than causal, and the observed SDI-burden patterns should be interpreted accordingly. The analytic workflow consisted of extracting the GBD outputs, restructuring them by location and year, calculating age-standardized rates and temporal changes, and then fitting the spline models.”

Results

Comparisons among Iran, MENA, and global estimates remain descriptive without formal comparative analyses.

Response: We agree that the comparisons across Iran, MENA, and global estimates are descriptive and were not based on formal hypothesis testing or pairwise comparative modeling. To avoid overinterpretation, we revised the text to present these findings as contextual comparisons rather than statistically tested differences.

Provincial variation is described extensively but remains observational and lacks spatial analytical methods.

Response: The provincial analysis in this study is descriptive and ecological in nature, based on GBD subnational estimates. We clarified this point in the Results and avoided language that might imply spatial clustering or spatial inference beyond the available data.

Age-related prevalence findings require cautious interpretation because prevalence in congenital conditions accumulates over time and may not reflect disease biology.

Response: We appreciate this important point. We revised the age-related section to avoid implying that prevalence patterns directly reflect disease occurrence at a given age. Instead, we clarified that prevalence in a congenital condition reflects accumulation over time, survival, and access to care, and therefore should be interpreted cautiously.

The higher DALY burden among females under five years is reported without exploration of possible explanations.

Response: We agree that this finding should be presented cautiously. Because the current analysis was descriptive, we did not attempt causal explanation in the Results. We revised the text to report the pattern without overinterpreting it and noted that possible explanations are better considered in the Discussion.

Discussion

Several interpretations attribute declining burden to improved surgical care, healthcare access, neonatal support, and health-system improvements despite these variables not being directly analyzed.

Response: We revised the Discussion to make clear that these factors are plausible explanations for the observed decline, but they were not directly measured in this study. We therefore changed the wording to present them as cautious interpretations rather than conclusions.

Explanations regarding referral patterns, healthcare inequalities, and reporting differences remain speculative.

Response: The revised text now identifies referral patterns, differential case ascertainment, and reporting completeness as possible contributors to provincial variation, while explicitly noting that these are hypotheses rather than directly tested explanations.

The discussion occasionally shifts from epidemiologic interpretation toward causal inference unsupported by presented data.

Response: We appreciate this important comment. We revised the Discussion to maintain an epidemiologic and descriptive focus throughout, removed causal language, and ensured that the interpretation remains aligned with the ecological and modeled nature of the data.

The markedly larger reduction in DALYs observed in Iran relative to global estimates warrants deeper discussion.

Response: We expanded the Discussion to compare Iran’s reduction with global and MENA trends more explicitly and to note that the larger decline may reflect a combination of improved survival, changes in disability patterns, and modeled estimation differences. We also clarified that this comparison should be interpreted cautiously because the estimates come from different geographic levels and modeling contexts. It reads:

“The larger decline in DALYs observed in Iran compared with global estimates (4) may reflect a combination of improving survival, changes in disability patterns, and differences in model-based estimation across geographic levels. However, this interpretation remains cautious because these factors were not directly tested in the present analysis (15).”

Ecological SDI findings should be interpreted cautiously to avoid ecological fallacy.

Response: As suggested, we revised the text to state clearly that the SDI analysis is ecological and descriptive, and that it cannot be interpreted as evidence of individual-level socioeconomic effects. It reads:

“This ecological association should be interpreted cautiously to avoid ecological fallacy, because province-level SDI does not necessarily represent the socioeconomic conditions of individual patients.”

Limitations

Important limitations are incompletely addressed.

Response: We agree and expanded the limitations substantially.

The study should explicitly discuss reliance on modeled rather than observed data.

Response: We added a direct statement that the analysis relies on GBD modeled estimates rather than primary observed registry data, and that this is an important limitation.

Limitations related to sparse provincial data, ecological analysis, inability to evaluate subtype-specific patterns, and inability to distinguish syndromic and non-syndromic OFCs should be acknowledged.

Response: We have added these limitations explicitly and noted that they constrain interpretation of provincial differences and prevent phenotype-specific and etiologic conclusions. It reads:

“Third, we could not evaluate the influence of specific risk factors at the provincial level within the GBD framework, so causal inferences about drivers of observed trends are limited. Fourth, because OFCs were analyzed as a combined category, we could not evaluate subtype-specific patterns for cleft lip with or without palate versus isolated cleft palate. Fifth, the GBD framework does not allow us to distinguish syndromic from non-syndromic OFCs, which limits etiologic interpretation. Sixth, the provincial and SDI analyses are ecological and should not be interpreted at the individual level. Nevertheless, the use of the most recent, internationally comparable GBD 2023 framework to produce a comprehensive, subnational picture of OFC burden for Iran across three decades provides useful context for understanding long-term trends and provincial inequalities. Future studies should compare GBD-derived estimates with Iranian congenital anomaly registry and hospital-based data when such data become available.”

Conclusion

The conclusion contains policy recommendations that extend beyond the presented analyses.

Response: We toned down the conclusion to focus more closely on the findings actually demonstrated in the study.

Statements regarding prevention strategies and healthcare interventions should be more closely aligned with observed epidemiological findings.

Response: We revised the conclusion to emphasize burden reduction, inequities, and the need for stronger surveillance and access to care, while avoiding overly specific preventive claims not directly supported by the analysis. It reads”

“Iran has experienced declines in OFC burden from 1990 to 2023, while national prevalence has remained relatively stable and provincial and age-specific disparities persist. The highest disability burden was among young children and was inversely associated with SDI, highlighting ongoing equity gaps in access to timely and comprehensive cleft care. These findings highlight persistent burden and inequity patterns that may inform future planning for surveillance, diagnosis, treatment access, and follow-up care. More specific prevention and health-system recommendations should be interpreted in light of future studies that directly examine risk factors, care pathways, and service availability.”

Figures and Tables

Figure 1 is visually crowded

Attachment Submitted filename: RTR PLOS ONE-Orofacial cleft.doc

# Peer review: Decision Letter 1

**DOI:** 10.1371/journal.pone.0352804.r003

**Authors:** Omid Beiki, Omid Beiki

12 Jun 2026

PONE-D-26-17844R1

The burden and epidemiology of orofacial clefts in Iran from 1990 to 2023: a Global Burden of Disease study with subnational socio-demographic index analysis

PLOS One

Dear Dr. Bakhtiari,

Thank you for submitting your manuscript to PLOS ONE. After careful consideration, we feel that it has merit but does not fully meet PLOS ONE’s publication criteria as it currently stands. Therefore, we invite you to submit a revised version of the manuscript that addresses the points raised during the review process.

Please submit your revised manuscript by Jul 27 2026 11:59PM. If you will need more time than this to complete your revisions, please reply to this message or contact the journal office at plosone@plos.org. When you're ready to submit your revision, log on to https://www.editorialmanager.com/pone/ and select the 'Submissions Needing Revision' folder to locate your manuscript file.

Please include the following items when submitting your revised manuscript:

- A letter that responds to each point raised by the academic editor and reviewer(s). You should upload this letter as a separate file labeled 'Response to Reviewers'.
- A marked-up copy of your manuscript that highlights changes made to the original version. You should upload this as a separate file labeled 'Revised Manuscript with Track Changes'.
- An unmarked version of your revised paper without tracked changes. You should upload this as a separate file labeled 'Manuscript'.

If you would like to make changes to your financial disclosure, please include your updated statement in your cover letter. Guidelines for resubmitting your figure files are available below the reviewer comments at the end of this letter.

If applicable, we recommend that you deposit your laboratory protocols in protocols.io to enhance the reproducibility of your results. Protocols.io assigns your protocol its own identifier (DOI) so that it can be cited independently in the future. For instructions see: https://journals.plos.org/plosone/s/submission-guidelines#loc-laboratory-protocols. Additionally, PLOS ONE offers an option for publishing peer-reviewed Lab Protocol articles, which describe protocols hosted on protocols.io. Read more information on sharing protocols at https://plos.org/protocols?utm_medium=editorial-email&utm_source=authorletters&utm_campaign=protocols.

As the corresponding author, your ORCID iD is verified in the submission system and will appear in the published article. PLOS supports the use of ORCID, and we encourage all coauthors to register for an ORCID iD and use it as well. Please encourage your coauthors to verify their ORCID iD within the submission system before final acceptance, as unverified ORCID iDs will not appear in the published article. *Only*  the individual author can complete the verification step; PLOS staff *cannot*  verify ORCID iDs on behalf of authors.

We look forward to receiving your revised manuscript.

Kind regards,

Omid Beiki, M.D., Ph.D.

Academic Editor

PLOS One

Journal Requirements:

1. If the reviewer comments include a recommendation to cite specific previously published works, please review and evaluate these publications to determine whether they are relevant and should be cited. There is no requirement to cite these works unless the editor has indicated otherwise.

2. Please review your reference list to ensure that it is complete and correct. If you have cited papers that have been retracted, please include the rationale for doing so in the manuscript text, or remove these references and replace them with relevant current references. Any changes to the reference list should be mentioned in the rebuttal letter that accompanies your revised manuscript. If you need to cite a retracted article, indicate the article’s retracted status in the References list and also include a citation and full reference for the retraction notice.

[Note: HTML markup is below. Please do not edit.]

Reviewer's Responses to Questions

**Comments to the Author**

1. If the authors have adequately addressed your comments raised in a previous round of review and you feel that this manuscript is now acceptable for publication, you may indicate that here to bypass the “Comments to the Author” section, enter your conflict of interest statement in the “Confidential to Editor” section, and submit your "Accept" recommendation.

Reviewer #1: All comments have been addressed

Reviewer #2: (No Response)

**********

2. Is the manuscript technically sound, and do the data support the conclusions?

The manuscript must describe a technically sound piece of scientific research with data that supports the conclusions. Experiments must have been conducted rigorously, with appropriate controls, replication, and sample sizes. The conclusions must be drawn appropriately based on the data presented.

Reviewer #1: Yes

Reviewer #2: Yes

**********

3. Has the statistical analysis been performed appropriately and rigorously?

Reviewer #1: Yes

Reviewer #2: Yes

**********

4. Have the authors made all data underlying the findings in their manuscript fully available?

The PLOS Data policy requires authors to make all data underlying the findings described in their manuscript fully available without restriction, with rare exception (please refer to the Data Availability Statement in the manuscript PDF file). The data should be provided as part of the manuscript or its supporting information, or deposited to a public repository. For example, in addition to summary statistics, the data points behind means, medians and variance measures should be available. If there are restrictions on publicly sharing data—e.g. participant privacy or use of data from a third party—those must be specified.

Reviewer #1: Yes

Reviewer #2: (No Response)

**********

5. Is the manuscript presented in an intelligible fashion and written in standard English?

PLOS ONE does not copyedit accepted manuscripts, so the language in submitted articles must be clear, correct, and unambiguous. Any typographical or grammatical errors should be corrected at revision, so please note any specific errors here.

Reviewer #1: Yes

Reviewer #2: Yes

**********

6. Review Comments to the Author

Please use the space provided to explain your answers to the questions above. You may also include additional comments for the author, including concerns about dual publication, research ethics, or publication ethics. (Please upload your review as an attachment if it exceeds 20,000 characters)

Reviewer #1: The authors have addressed my comments satisfactorily and significantly improved the manuscript. The revised version is clearer, more balanced, and methodologically transparent.

Reviewer #2: 1. The manuscript should clearly distinguish whether the authors conducted any independent modeling or simply analyzed pre-existing IHME/GBD outputs. As currently written, this distinction is blurred. It needs to explicitly state that no new primary DisMod-MR 2.1 modeling was performed, and that the work is based on descriptive analysis of extracted GBD data rather than reproduction of the original modeling framework.

2. The aggregation of all orofacial clefts (ICD-10 Q35–Q37) into a single category conceals meaningful clinical and biological differences between subtypes such as cleft palate and cleft lip with or without cleft palate. While this grouping may be constrained by the structure of available GBD data, the manuscript should acknowledge that it reduces clinical granularity and may limit the interpretability and real-world applicability of burden estimates.

3. The use of smoothing splines to model the relationship between SDI and burden metrics is not sufficiently described. Key details such as smoothing parameters, degrees of freedom, and model fit assessment are missing. The analysis should be explicitly framed as an exploratory, descriptive approach rather than inferential modeling, with clearer justification of how curve smoothness was selected to avoid the impression of arbitrary fitting.

4. The observed inverse association between provincial SDI and DALYs is interpreted too strongly in places, risking ecological fallacy. Because the data are aggregated at the provincial level, these findings should not be extended to individual-level socioeconomic risk. The results should be framed strictly as area-level patterns rather than implying individual-level relationships.

5. The manuscript highlights stable prevalence over time alongside a substantial reduction in DALYs and mortality, but this pattern is not well explained. A clearer interpretation is needed, likely involving improved survival, better long-term management, and the accumulation of treated individuals who continue to live with the condition, which maintains prevalence even as severity and mortality decline.

6. Several causal explanations for declining DALYs and mortality are stated too definitively. Since the GBD dataset does not directly measure healthcare access, prenatal screening, or underlying risk factors, these should be reframed as possible contributing hypotheses rather than confirmed drivers of change.

7. The policy recommendations extend beyond what can be justified by a descriptive epidemiological analysis. Specific clinical or preventive interventions should be avoided. Instead, conclusions should focus on higher-level public health implications such as strengthening congenital anomaly surveillance systems, improving registry completeness, and supporting broad resource allocation and health system planning.

**********

7. PLOS authors have the option to publish the peer review history of their article (what does this mean?). If published, this will include your full peer review and any attached files.

If you choose “no”, your identity will remain anonymous but your review may still be made public.

**Do you want your identity to be public for this peer review?**  For information about this choice, including consent withdrawal, please see our Privacy Policy.

Reviewer #1: No

Reviewer #2: No

**********

[NOTE: If reviewer comments were submitted as an attachment file, they will be attached to this email and accessible via the submission site. Please log into your account, locate the manuscript record, and check for the action link "View Attachments". If this link does not appear, there are no attachment files.]

To ensure your figures meet our technical requirements, please review our figure guidelines: https://journals.plos.org/plosone/s/figures

You may also use PLOS’s free figure tool, NAAS, to help you prepare publication quality figures: https://journals.plos.org/plosone/s/figures#loc-tools-for-figure-preparation.

NAAS will assess whether your figures meet our technical requirements by comparing each figure against our figure specifications.

# Author comment: Author response to Decision Letter 2

**DOI:** 10.1371/journal.pone.0352804.r004

13 Jun 2026

Response to Reviewers:

Manuscript reference number: PONE-D-26-17844R1

The burden and epidemiology of orofacial clefts in Iran from 1990 to 2023: a Global Burden of Disease study with subnational socio-demographic index analysis

Dear Dr. Beiki,

Thank you for giving us the opportunity to revise our paper for potential publication in PLOS ONE. We are grateful to the reviewers and the Editors for their constructive and positive comments on our paper. We have addressed each of the comments in our revision, which has further improved the article. Below we detail our changes and respond to each of the comments in turn.

Reviewer Comments to Author:

Reviewer: 1

Comments to Author

The authors have addressed my comments satisfactorily and significantly improved the manuscript. The revised version is clearer, more balanced, and methodologically transparent.

Response: Thank you very much for reviewing the revised manuscript and your positive feedback.

Reviewer: 2

1. The manuscript should clearly distinguish whether the authors conducted any independent modeling or simply analyzed pre-existing IHME/GBD outputs. As currently written, this distinction is blurred. It needs to explicitly state that no new primary DisMod-MR 2.1 modeling was performed, and that the work is based on descriptive analysis of extracted GBD data rather than reproduction of the original modeling framework.

Response: Thank you so much for your time to review the revised manuscript and your valuable comments. We have revised the Methods to state that this study is based on a descriptive secondary analysis of finalized GBD 2023 outputs and that no new primary DisMod-MR 2.1 modeling was performed by the authors. We also clarified that the manuscript does not reproduce the original IHME modeling process but instead summarizes and interprets the available estimates for Iran and its provinces. It reads:

“We extracted the finalized GBD 2023 estimates and conducted a descriptive secondary analysis of these pre-generated outputs. No new primary DisMod-MR 2.1 modeling was performed in this study.”

“We did not independently re-fit the full GBD model. Instead, we extracted and analyzed the finalized GBD 2023 estimates as a descriptive secondary analysis.”

2. The aggregation of all orofacial clefts (ICD-10 Q35–Q37) into a single category conceals meaningful clinical and biological differences between subtypes such as cleft palate and cleft lip with or without cleft palate. While this grouping may be constrained by the structure of available GBD data, the manuscript should acknowledge that it reduces clinical granularity and may limit the interpretability and real-world applicability of burden estimates.

Response: We appreciate this point and agree that combining all OFC phenotypes into a single category reduces clinical granularity. We revised the manuscript to explain that this aggregation was necessary because the relevant GBD 2023 outputs were available only in combined form for Iran and its provinces. We also added text noting that this approach improves comparability across locations and years but limits phenotype-specific interpretation and real-world clinical applicability. It reads:

“Although OFC phenotypes differ in biological and clinical characteristics, they were analyzed as a combined category because this is how the GBD 2023 outputs were available for Iran and its provinces. This grouping improves comparability across years and locations, but it reduces clinical granularity and limits phenotype-specific interpretation and real-world applicability of the burden estimates. The equal-distribution assumption among sequelae was used within the GBD framework when more granular sequela-specific data were unavailable, and we acknowledge that this simplifying assumption may influence DALY estimation.”

3. The use of smoothing splines to model the relationship between SDI and burden metrics is not sufficiently described. Key details such as smoothing parameters, degrees of freedom, and model fit assessment are missing. The analysis should be explicitly framed as an exploratory, descriptive approach rather than inferential modeling, with clearer justification of how curve smoothness was selected to avoid the impression of arbitrary fitting.

Response: We appreciate your suggestion and have expanded the description of the SDI analysis in the Methods. We emphasize that the smoothing spline analysis was intended as an exploratory visualization of the relationship between SDI and age-standardized OFC burden metrics and was not designed as an inferential or predictive model. The spline curves were fitted using standard smoothing spline procedures in R, with the smoothing parameter selected automatically through generalized cross-validation, which balances model fit against complexity and helps avoid overfitting. We did not pre-specify the degrees of freedom or manually tune the smoothness parameter, as the objective was descriptive visualization rather than formal hypothesis testing or model optimization. Consequently, we did not perform additional assessments of model fit or sensitivity analyses, and the resulting curves should be interpreted as graphical summaries of non-linear ecological patterns rather than evidence of causal or statistically significant associations. It reads:

“Spline curves were fitted in R using standard smoothing spline procedures, with the smoothing parameter selected automatically by generalized cross-validation, allowing the algorithm to balance goodness of fit against model complexity and reduce the risk of overfitting. No degrees of freedom or smoothing parameters were manually specified or optimized, because the analysis was intended to provide descriptive visualization rather than formal statistical inference or prediction. Accordingly, no hypothesis testing or model-based causal interpretation was performed, and the observed SDI–burden relationships should be interpreted as population-level ecological patterns rather than individual-level associations.”

4. The observed inverse association between provincial SDI and DALYs is interpreted too strongly in places, risking ecological fallacy. Because the data are aggregated at the provincial level, these findings should not be extended to individual-level socioeconomic risk. The results should be framed strictly as area-level patterns rather than implying individual-level relationships.

Response: We revised the Results and Discussion to make clear that the inverse association between provincial SDI and DALY burden is an ecological, province-level pattern and should not be interpreted as evidence of individual-level socioeconomic risk. We also noted the risk of ecological fallacy and avoided wording that could imply causality at the individual level. It reads:

“The burden of OFCs, represented by the age-standardized DALY rate per 100,000 population, showed an inverse area-level association with the SDI.”

“Because these findings are based on ecological, modeled estimates, they describe population-level patterns rather than individual-level associations.”

“This ecological association should be interpreted cautiously to avoid ecological fallacy, because province-level SDI does not necessarily represent the socioeconomic conditions of individual patients.”

5. The manuscript highlights stable prevalence over time alongside a substantial reduction in DALYs and mortality, but this pattern is not well explained. A clearer interpretation is needed, likely involving improved survival, better long-term management, and the accumulation of treated individuals who continue to live with the condition, which maintains prevalence even as severity and mortality decline.

Response: We expanded the Discussion to better explain why prevalence may remain stable even when DALYs and mortality decline. The revised text now notes that in a congenital condition such as OFC, prevalence may accumulate across age groups because treated individuals survive longer, while disability burden and mortality may decrease with improved survival, earlier management, and better long-term care. This interpretation is now stated more clearly and cautiously. It reads:

“The decline in DALYs and deaths is consistent with a broader improvement in population health and may reflect improved survival, better long-term management, and reduced severity among affected individuals over time, but our study cannot determine the exact reason (22). In particular, in a congenital condition such as OFC, prevalence may remain stable because treated individuals continue to survive into later age groups, even as mortality and disability burden decline. Moreover, population health gains such as reductions in infant mortality and improved neonatal services can reduce OFC-related morbidity (23). Similarly, observed provincial inequalities may reflect differences in access, referral, ascertainment, and service availability, but the present analysis does not allow these mechanisms to be distinguished (18, 19). Accordingly, these factors should be considered plausible hypotheses rather than confirmed determinants of the observed trends.”

6. Several causal explanations for declining DALYs and mortality are stated too definitively. Since the GBD dataset does not directly measure healthcare access, prenatal screening, or underlying risk factors, these should be reframed as possible contributing hypotheses rather than confirmed drivers of change.

Response: We revised the Discussion to frame surgical advances, neonatal care, healthcare access, and system improvements as possible contributing hypotheses rather than confirmed drivers of change. We also added explicit caution that these factors were not directly measured in the present analysis, so causal inference is not possible from the current dataset. It reads:

“The larger decline in DALYs observed in Iran compared with global estimates (4) might be partially due to a combination of improving survival, changes in disability patterns, and differences in model-based estimation across geographic levels. However, this interpretation remains cautious because these factors were not directly tested in the present analysis. Also, these should be considered as possible contributing hypotheses rather than confirmed drivers of change (15).”

7. The policy recommendations extend beyond what can be justified by a descriptive epidemiological analysis. Specific clinical or preventive interventions should be avoided. Instead, conclusions should focus on higher-level public health implications such as strengthening congenital anomaly surveillance systems, improving registry completeness, and supporting broad resource allocation and health system planning.

Response: Thank you for your recommendations. We revised the conclusion to focus on the observed burden patterns, provincial disparities, and the implications for surveillance, registry completeness, resource allocation, and health-system planning. We removed or softened recommendations that went beyond what can be directly supported by the GBD data. It reads:

“Iran has experienced declines in OFC burden from 1990 to 2023, while national prevalence has remained relatively stable and provincial and age-specific disparities persist. The highest disability burden was among young children and was inversely associated with SDI, highlighting ongoing equity gaps in access to timely and comprehensive cleft care. These findings highlight persistent burden and inequity patterns that may inform future planning for surveillance, registry completeness, diagnosis, treatment access, and follow-up care. More specific prevention and health-system recommendations should be interpreted in light of future studies that directly examine risk factors, care pathways, and service availability.”

Attachment Submitted filename: R2 RTR PLOS ONE-Orofacial cleft.doc

# Editor report: Decision Letter 2

**DOI:** 10.1371/journal.pone.0352804.r005

**Authors:** Omid Beiki, Omid Beiki, Omid Beiki

15 Jun 2026

The burden and epidemiology of orofacial clefts in Iran from 1990 to 2023: a Global Burden of Disease study with subnational socio-demographic index analysis

PONE-D-26-17844R2

Dear Dr. Bakhtiari,

We’re pleased to inform you that your manuscript has been judged scientifically suitable for publication and will be formally accepted for publication once it meets all outstanding technical requirements.

Within one week, you’ll receive an e-mail detailing the required amendments. When these have been addressed, you’ll receive a formal acceptance letter and your manuscript will be scheduled for publication.

An invoice will be generated when your article is formally accepted. Please note, if your institution has a publishing partnership with PLOS and your article meets the relevant criteria, all or part of your publication costs will be covered. Please make sure your user information is up-to-date by logging into Editorial Manager at Editorial Manager® and clicking the ‘Update My Information' link at the top of the page. For questions related to billing, please contact billing support.

If your institution or institutions have a press office, please notify them about your upcoming paper to help maximize its impact. If they’ll be preparing press materials, please inform our press team as soon as possible -- no later than 48 hours after receiving the formal acceptance. Your manuscript will remain under strict press embargo until 2 pm Eastern Time on the date of publication. For more information, please contact onepress@plos.org.

Kind regards,

Omid Beiki, M.D., Ph.D.

Academic Editor

PLOS One

Additional Editor Comments (optional):

Reviewers' comments:

# Editor report: Acceptance letter

**DOI:** 10.1371/journal.pone.0352804.r006

**Authors:** Omid Beiki, Omid Beiki, Omid Beiki

PONE-D-26-17844R2

PLOS One

Dear Dr. Bakhtiari,

I'm pleased to inform you that your manuscript has been deemed suitable for publication in PLOS One. Congratulations! Your manuscript is now being handed over to our production team.

At this stage, our production department will prepare your paper for publication. This includes ensuring the following:

* All references, tables, and figures are properly cited

* All relevant supporting information is included in the manuscript submission,

* There are no issues that prevent the paper from being properly typeset

You will receive further instructions from the production team, including instructions on how to review your proof when it is ready. Please keep in mind that we are working through a large volume of accepted articles, so please give us a few days to review your paper and let you know the next and final steps.

Lastly, if your institution or institutions have a press office, please let them know about your upcoming paper now to help maximize its impact. If they'll be preparing press materials, please inform our press team within the next 48 hours. Your manuscript will remain under strict press embargo until 2 pm Eastern Time on the date of publication. For more information, please contact onepress@plos.org.

You will receive an invoice from PLOS for your publication fee after your manuscript has reached the completed accept phase. If you receive an email requesting payment before acceptance or for any other service, this may be a phishing scheme. Learn how to identify phishing emails and protect your accounts at https://explore.plos.org/phishing.

If we can help with anything else, please email us at customercare@plos.org.

Thank you for submitting your work to PLOS ONE and supporting open access.

Kind regards,

PLOS ONE Editorial Office Staff

on behalf of

Dr. Omid Beiki

Academic Editor

PLOS One
